# Subterranean synergies: termite bacterial diversity and eugenol-mediated selective dysbiosis

**DOI:** 10.3389/fmicb.2026.1818254

**Published:** 2026-06-17

**Authors:** Arnab Purohit, Amrita Chakraborty, Jan Křivánek, Robert Hanus, Keerthana Mohan, Amit Roy

**Affiliations:** 1 Faculty of Forestry and Wood Sciences, Czech University of Life Sciences, Prague, Czechia; 2 Institute of Organic Chemistry and Biochemistry of the Czech Academy of Sciences, Prague, Czechia

**Keywords:** amplicon sequencing, *Coptotermes formosanus*, eugenol, LD50, *Reticulitermes flavipes*, sublethal, subterranean termites

## Abstract

Subterranean termites, *Coptotermes formosanus* and *Reticulitermes flavipes* (Isoptera: Rhinotermitidae) rank among the most economically significant wood-feeding pests, relying on complex symbiotic associations with gut microbes to facilitate lignocellulose digestion, nitrogen fixation, and other essential metabolic processes. Although their bacterial communities have been individually described, direct comparisons and the effects of plant-derived bioactive compounds on these symbioses remain poorly understood. Here, we present the first comparative analysis of bacterial communities in these two termite species under phytochemical stress induced by eugenol, a phenolic monoterpenoid with known insecticidal and antimicrobial properties. Using 16S rRNA amplicon sequencing, we demonstrate that although the two species harbor distinct bacterial assemblages, they share a conserved core microbiota dominated by Spirochaetota. *C. formosanus* harbored a higher relative abundance of Bacteroidota, whereas *R. flavipes* exhibited prevalence of Firmicutes, Elusimicrobiota, and Actinobacteria. Despite these differences, both species shared a core bacterial community dominated by Spirochaetota. Eugenol exposure resulted in significant termite mortality and induced taxon-specific shifts in bacterial composition without altering overall community diversity, indicating a selective restructuring rather than a broad-spectrum disruption of the termite bacteriome. Specifically, eugenol decreased the abundance of Spirochaetota, particularly the genus *Treponema*, while enriching Firmicutes and Proteobacteria. This pattern of selective dysbiosis indicates a mechanistic shift away from non-specific antimicrobial effects, underscoring targeted microbial restructuring as a key ecological consequence of eugenol exposure. Moreover, PICRUST2-based predictions indicated that eugenol treatment alters microbial functional potential, including pathways associated with carbohydrate metabolism, fermentation, and amino acid biosynthesis, suggesting that eugenol selectively interferes with key symbiotic functions critical to termite survival. These findings demonstrate species-specific differences in termite-associated bacterial assemblages and highlight the potential of eugenol to selectively disrupt functionally important microbial taxa, providing a foundation for microbiome-targeted, environmentally sustainable termite control strategies.

## 1 Introduction

Termites (Isoptera) represent one of the earliest social insect lineages, with a remarkable history spanning over 130 million years (Korb, 2007; Bourguignon et al., 2014). They play a crucial ecological role by decomposing and recycling wood and plant materials, improving soil structure and fertility, reducing greenhouse gas emissions, and contributing to habitat formation (Korb, 2007). However, their feeding habit often causes extensive damage to wooden structures and crops, resulting in substantial financial losses and inconvenience in human settlements (Rouland-Lefèvre, 2011). The global economic toll caused by termite damage was estimated at a staggering US$40 billion in 2010 (Rust and Su, 2012). Among over 3000 termite species described worldwide, 183 have been identified as pests. Subterranean termites (Isoptera: Rhinotermitidae), including *Coptotermes formosanus* and *Reticulitermes flavipes*, are wood-feeding lower termites that cause extensive structural damage to wooden structures across widespread geographic regions (Perdereau et al., 2019; Lee et al., 2021). The ecological success of lower termites is tightly linked to their mutualistic symbiosis with gut microorganisms. Although termites produce endogenous cellulases from the midgut epithelium and labial glands, efficient lignocellulose degradation depends largely on their gut microbiota, composed primarily of bacteria and protists (Ni and Tokuda, 2013). Several studies reported the pivotal role of bacterial symbionts in efficient cellulose digestion in the termite gut via acetate metabolism, hemicellulose and aromatic compound degradation, and nitrogen cycling (Hongoh et al., 2008; Lucey and Leadbetter, 2014). Perturbations to these microbial communities, for example, reductions in bacterial abundance and diversity following antimicrobial treatments, have been directly associated with impaired lignocellulolytic activity (Peterson et al., 2015). Thus, termite fitness and colony persistence are fundamentally dependent on their gut microbial consortia.

Despite this central role of the microbiome in termite biology, most control strategies do not explicitly target these symbiotic associations. Current termite control strategies rely predominantly on synthetic termiticides and baiting systems, which face persistent challenges including environmental contamination, non-target toxicity, development of resistance, and limited long-term efficacy against socially resilient subterranean termite colonies (Togaev et al., 2024). Moreover, subterranean termites exhibit cryptic nesting behavior, large colony sizes, and remarkable social resilience, enabling colonies to recover even after substantial worker mortality (Su and Scheffrahn, 1998). Behavioral avoidance and reduced bait acceptance further limit the long-term effectiveness of baiting systems, particularly under field conditions (Togaev et al., 2024). These limitations highlight the need for innovative strategies that move beyond direct toxicity and instead exploit biological vulnerabilities, such as termite–microbe dependencies.

In this context, plant-derived biopesticides have gained increasing recognition as environmentally sustainable alternatives for managing subterranean termites (Mishra et al., 2021; Gupta et al., 2023; Mogilicherla et al., 2023). Among these, eugenol (4-allyl-2-methoxyphenol), a phenolic monoterpenoid found in clove, cinnamon, basil, etc., has received significant attention due to its diverse biological activities, notably its antimicrobial (Qian et al., 2020; Elbestawy et al., 2023; Olaimat et al., 2024) and insecticidal properties (Cornelius et al., 1997; Enan, 2001; Huang et al., 2002; Xie et al., 2015). Eugenol, the major phenylpropanoid of clove oil, exhibits broad-spectrum antimicrobial activity against bacteria and fungi, including both Gram-positive and Gram-negative bacteria (Cui et al., 2018; Maggini et al., 2024). As a hydrophobic and lipophilic phenolic compound, eugenol can interact with microbial membranes, disrupting membrane integrity, increasing permeability, and leading to leakage of intracellular contents, which may deplete cytosolic molecules necessary for bacterial metabolism and survival (Gill and Holley, 2006). In addition to membrane disruption, eugenol has been reported to interfere with quorum sensing in *P. aeruginosa and E. coli* (Zhou et al., 2013). Its antimicrobial activity may also involve enzyme inhibition, as the hydroxyl group of eugenol has been suggested to bind and inhibit enzymes such as protease, histidine carboxylase, and amylase in *Enterobacter aerogenes* (Devi et al., 2010; Marchese et al., 2017). Furthermore, eugenol has been reported to act synergistically with conventional antimicrobials and may induce intracellular reactive oxygen species, contributing to growth inhibition, membrane damage, DNA damage, and ultimately cell death (Hyldgaard et al., 2012). Furthermore, an *in vitro* study on pig gut microflora revealed that eugenol showed antimicrobial activity in the midgut (jejunum), reducing the bacterial abundance (Michiels et al., 2009). Beyond its direct antimicrobial effects, eugenol also demonstrates pronounced insecticidal efficacy across diverse insect taxa, suggesting that its biological activity may extend to both insect hosts and their associated microbes. For example, studies on *Musca domestica* have shown that eugenol can cause significant mortality in immature stages and induce developmental abnormalities at sublethal concentrations (da Silva et al., 2020). Additionally, eugenol derivatives have shown enhanced insecticidal activity against *Spodoptera frugiperda* (fall armyworm) (Coelho et al., 2022). Importantly, its effectiveness against termites has also been documented (Cornelius et al., 1997; Yang et al., 2023). Notably, its eco-friendly nature and low mammalian toxicity make eugenol an appealing candidate for combating subterranean termites (Cornelius et al., 1997). However, the practical application of eugenol for termite control may be limited by its high volatility and susceptibility to oxidative degradation, which reduces its persistence under field conditions. Encapsulation at micro- or nano-scales offers a promising strategy to enhance eugenol stability and enable controlled release, thereby improving its insecticidal efficacy and durability (Ayllón-Gutiérrez et al., 2024). Importantly, beyond its direct insecticidal properties, eugenol exhibits broad-spectrum antimicrobial activity. Antimicrobial properties of eugenol suggest a potential impact on the insect microbiome (Marchese et al., 2017; Guimarães et al., 2019). Given the pivotal role of gut symbionts in termite nutrition, immunity, and overall fitness, disruption of microbial homeostasis may critically undermine termite survival. Yet, the extent to which eugenol alters termite-associated bacterial communities remains insufficiently characterized. Most termite control studies evaluate mortality endpoints without examining microbiome dynamics, thereby overlooking a key mechanistic dimension. Given that termite survival depends on complex microbial networks that enable cellulose digestion and nutrient cycling, chemically induced dysbiosis could critically compromise colony-level function. A deeper understanding of these microbial responses is therefore essential for the rational design of microbiome-informed control strategies.

Despite extensive research on conventional termite control strategies and the growing interest in plant-derived compounds such as eugenol, a key knowledge gap remains in understanding how these compounds influence termite-associated microbial symbioses. In particular, it is unclear whether phytochemicals exert direct, selective effects on specific microbial taxa or whether observed changes in the microbiome arise indirectly through host physiological stress or altered gut conditions. Addressing this distinction is critical, as termite survival is closely linked to the functional integrity of its gut microbiota. In this context, the present study compares the bacterial communities associated with *C. formosanus* and *R. flavipes* using high-throughput sequencing of the 16S rRNA gene to identify species-specific patterns in bacterial assemblages. To our knowledge, this work represents the first comparative analysis of the bacterial communities of the two subterranean termite species under phytochemical stress. We further evaluate the effects of sublethal and lethal doses of eugenol exposure on termite mortality and bacterial community structure. We hypothesize that eugenol, as a xenobiotic compound, induces taxon-specific shifts in bacterial communities rather than broad-spectrum disruption and potentially impacts host health and survival. While this study does not aim to serve as a field application trial, it advances mechanistic understanding of termite-microbe interactions. By examining bacterial dynamics under both control and chemically stressed conditions, the study provides a conceptual basis for microbiome-mediated, environmentally sustainable approaches to termite management.

## 2 Materials and methods

### 2.1 Termite collection, experimental setup, and dose determination

Experimental termites were extracted from laboratory colonies of *C. formosanus* and *R. flavipes* (Heterotermitidae), reared in dark air-conditioned rooms at 25°C and 75–80% relative humidity in glass containers containing regularly moistened sand as substrates and fed spruce wood slices. The mortality experiments were carried out in plastic Petri dishes (5.5 cm diameter) lined with Watmann 1 filter papers (Hrdı et al., 2006) and groups of 40 workers + 5 or 1 soldier (*C. formosanus* and *R. flavipes*, respectively) were placed in the dishes. The filter papers were impregnated with different doses of eugenol dissolved in 100 μL of solvent (*n*-hexane). Control groups were established using filter papers impregnated with 100 μL of *n*-hexane. After solvent evaporation, filter papers were remoistened with distilled water, dishes were sealed with parafilm, and termite survival was recorded at 12 h intervals over 120 h. The time-related survival data were transformed into survival curves, allowing the determination of sublethal doses at specific time points. The same data were then used to calculate LD50 eugenol doses using probit regression. The 48-h time point was selected for downstream sampling because it corresponded to the interval used to define the sublethal and LD50 doses and enabled consistent comparisons between the control and treated groups.

### 2.2 Termite samples for amplicon sequencing

From the above-mentioned data, we estimated the sublethal dose of 0.0625 mg for both species, and the LD50 dose of 0.150 mg for *C. formosanus* and 0.125 mg for *R. flavipes* at 48 h. We then repeated the experiment with three treatments, i.e., Control, Sublethal (SL), and LD50 eugenol doses, with 12 replicates per treatment (480 workers per treatment), and after 48 h, 144 workers from each treatment were randomly selected. The controls were denoted as CF.Control and RF.Control, respectively, while sublethal (SL) and lethal (LD50) eugenol treatment was marked as CF.SL and CF.LD50 for *C. formosanus*, and RF.SL and RF.LD50 for *R. flavipes*. For amplicon sequencing, only worker termites were randomly collected from each replicate, flash-frozen on dry ice as whole-body samples, and stored at –80°C until DNA extraction. Workers were selected because they represented the primary feeding caste in direct contact with the treated substrate, thereby providing the most biologically relevant and homogeneous samples for microbial community analysis.

### 2.3 DNA extraction, amplification, and sequencing for termite bacteriome

The eugenol-treated and untreated control termite samples were disinfected by preliminary washing with 70% ethanol for 1 min, followed by rinsing with sterile water. This step was repeated thrice to remove any exogenous contamination from the environment. Total DNA was extracted from four termites (whole body) per replicate. The samples were homogenized in a Mixer Mill MM400 (Retsch, Germany) using two sterile steel beads (3 mm) for 2 min at 30 oscillations/sec, and the Nucleospin soil DNA purification kit (Macherey Nagel, Germany) was then used for DNA extraction following the manufacturer’s protocol. A total of four replicates for each treatment were prepared. DNA was quantified via the Qubit High Sensitivity dsDNA assay kit in a Qubit Fluorometer (ThermoFisher Scientific, Germany). DNA quality assessment involved electrophoresis on a 1% agarose gel. The DNA samples were sent for sequencing to Novogene Sequencing Company, China.

For amplicon sequencing, DNA was diluted to 1 ng/μL in sterile water. The PCR amplification of V3-V4 region of the bacterial 16S rRNA gene was carried out using universal primers [341F (5’-CCTAYGGGRBGCASCAG-3’), 806R (5’-GGACTACNNGGGTATCTAAT-3’)] (Klindworth et al., 2013) and Phusion High-Fidelity PCR Master Mix (New England Biolabs). To prevent the possibility of contamination, no template control was included, similar to our previous studies (Chakraborty et al., 2023a). Equi-densities of the amplicons were separated on a 2% agarose gel, and bands were gel-purified using the Qiagen Gel Extraction Kit (Qiagen, Germany), and the sequencing library was constructed using NEBNext Ultra II DNA Library Prep Kit (Illumina). The constructed library was quantified using a Qubit Fluorometer and qPCR, with quality assessment via Agilent Bioanalyzer 2,100 system. The sequencing reaction was carried out on Illumina NovaSeq 6,000 platform to generate 250 bp paired-end reads.

### 2.4 Bioinformatic data analysis

#### 2.4.1 Processing of sequencing data and species annotation

Amplicon sequencing data were analyzed using QIIME2 2022.2 (Bolyen et al., 2019). FLASH (V1.2.11) eliminated barcodes and primer sequences and assembled the pair-end reads (Magoè and Salzberg, 2011).^1^ The “fastp” tool generated high-quality, clean tags from raw assembled tags, while VSEARCH identified and removed chimeras, providing effective tags for downstream analysis (Rognes et al., 2016). Further refinement excluded sequences with less than five read abundance, yielding amplicon sequence variants (ASVs) and a corresponding feature table using the DADA2 module in QIIME2 (ver 2022.2) (Callahan et al., 2016). SILVA (Release v138.1) database (Quast et al., 2012)^2^ and the Classify-sklearn module in QIIME2 (ver 2022.2) (Bokulich et al., 2018) facilitated ASV taxonomic annotation.

#### 2.4.2 α-diversity

α-Diversity indices evaluated bacterial community richness and diversity within samples. QIIME2 (ver 2022.2) calculated sequencing depth (Good’s coverage) (Good, 1953), bacterial richness (Chao1), evenness (Pielou), and diversity (Shannon) (Magurran and Magurran, 1988). The graphs were plotted in R software Version 2.15.3 (R Core Team, 2013), and Kruskal-Wallis test was performed to assess the statistical variations between groups for each α-diversity index.

To identify the core bacterial communities associated with each termite species, core bacteriome was defined as amplicon sequence variants (ASVs) present in at least 50% of samples within a given group. This prevalence-based threshold was selected to capture consistently occurring taxa while minimizing the inclusion of rare or sporadic members. Core ASVs were identified separately for *C. formosanus* and *R. flavipes* across all treatment groups. To assess the commonality and specificity of core bacteriome between the two termite species, shared and unique core ASVs were compared using presence-absence analysis.

#### 2.4.3 β-diversity

The UniFrac distance matrix (unweighted) calculated by QIIME2 (ver 2022.2) estimated variation in the bacterial diversity between samples (Lozupone et al., 2011). Non-metric multidimensional scaling (NMDS) and PCoA analysis were performed based on unweighted UniFrac and Bray-Curtis distances using the vegan package in R software Version 2.15.3 (Oksanen, 2011). To evaluate significant differences between sample communities, ADONIS analyses with 200 permutations in QIIME2 (ver 2022.2) evaluated significant differences between sample communities (Clarke, 1993; Anderson, 2001). ADONIS, a non-parametric multivariate variance test, analyses the sample group differences and their significance based on the UniFrac distance matrix (Stat et al., 2013). To detect significant variations in species abundance across groups, the MetaStat analysis applied multiple hypothesis tests for sparsely sampled features, and the false discovery rate (Paulson et al., 2011). Differences in species abundance were also evaluated using the *t*-test (*p* < 0.05) (D’Argenio et al., 2014). Identification of high-dimensional biomarkers for distinguishing tested samples was accomplished through Linear Discriminant Analysis Effect Size (LEfSe) analysis, with a predefined threshold set at linear discriminant analysis scores [LDA score (log10) > 4] (Segata et al., 2011).^3^

The putative functional profile of the identified bacteriome was derived using the PICRUSt2 software (Douglas et al., 2020). Predicting the metabolic functions of bacterial groups by mapping the sequenced bacterial group composition to different databases, including KEGG, MetaCyc, COG, and EC, provided putative functions associated with bacterial communities. The abundance data sourced from these databases was then used to generate heatmaps.

### 2.5 Quantitative PCR assay

The DNA extracted from two subterranean termites, with and without eugenol treatment (sublethal and lethal doses) as mentioned above, was used to estimate overall bacterial titer and the relative abundance of selected bacterial taxa across different eugenol treatments and control termites using quantitative PCR (qPCR). Three biological replicates (four termite whole bodies/replicates) were used for the qPCR assay. The overall bacterial titer was estimated using the standard curve with defined 16S rRNA gene copy numbers (Baños-Quintana et al., 2024). A standard curve was generated by first amplifying the target fragment, followed by purification and quantification of the PCR product. Then, eight serial 10-fold dilutions of the DNA concentrations were prepared to serve as standards. For each quantitative PCR (qPCR) reaction, 1 μL of the respective dilution was added to ensure consistent quantification across all reactions. The number of DNA copies in the standard dilutions was calculated, and subsequently, the efficiency of each qPCR reaction was estimated (Baños-Quintana et al., 2024). Furthermore, the relative abundance of the selected bacterial taxa (Bacteroidetes, Actinobacteria, and Firmicutes) was estimated using taxon-specific primers (Table 1). A reference primer was designed in-house targeting the elongation factor 1-alpha (EF-1α) gene (Table 1) for the two subterranean termites, *C. formosanus* and *R. flavipes*, and used to normalize qPCR data and determine fold change in bacterial abundance relative to the reference gene expression.

**TABLE 1:** Selected bacterial primers used for qPCR assay.

Target	Primer name	Primer sequence (5’–3’)	Amplicon length (bp)	Annealing temperature (°C)	References
Elongation Factor1-alpha	EF1α-F EF1α-R	AACCATCCTGGCCAGATTTC CAGTACGACGGTCACATTTCTC	109	60	In-house
Total bacteria	Eub338F Eub518R	ACT CCT ACG GGA GGC AGC AG ATT ACC GCG GCT GCT GG	180	60	(Thijs et al., 2017)
Actinobacteria	Actino235 Eub518	CGC GGC CTA TCA GCT TGT TG ATT ACC GCG GCT GCT GG	166	60	(Philippot et al., 2009)
Bacteroidetes	Cfb319 Eub518	GTA CTG AGA CAC GGA CCA ATT ACC GCG GCT GCT GG	181	60	(Philippot et al., 2009)
Firmicutes	Lgc353 Eub518	GCA GTA GGG AAT CTT CCG ATT ACC GCG GCT GCT GG	156	60	(Philippot et al., 2009)

The qPCR assay was performed using 4 μL template DNA (10 ng/μL), 5 μL SYBR Green PCR Master Mix (Applied Biosystems), and 0.5 μL each of forward and reverse primers (10 μM) (Table 1). The amplification was carried out at an initial denaturation step at 95°C for 5 min, followed by 40 cycles consisting of 95°C for 15 sec and annealing at 60°C for 30 s. Relative quantification (RQ) of the targeted bacterial populations was performed using the delta-delta Ct [2^∧^(-ΔΔCt)] method. Here, ΔCt represents the difference in threshold cycle (Ct) values between the bacterial-specific primers and the reference gene. The resulting 2^∧^(-ΔΔCt) value indicates the fold change in bacterial abundance relative to the reference gene.

## 3 Results

### 3.1 Termite mortality

**FIGURE 1:**
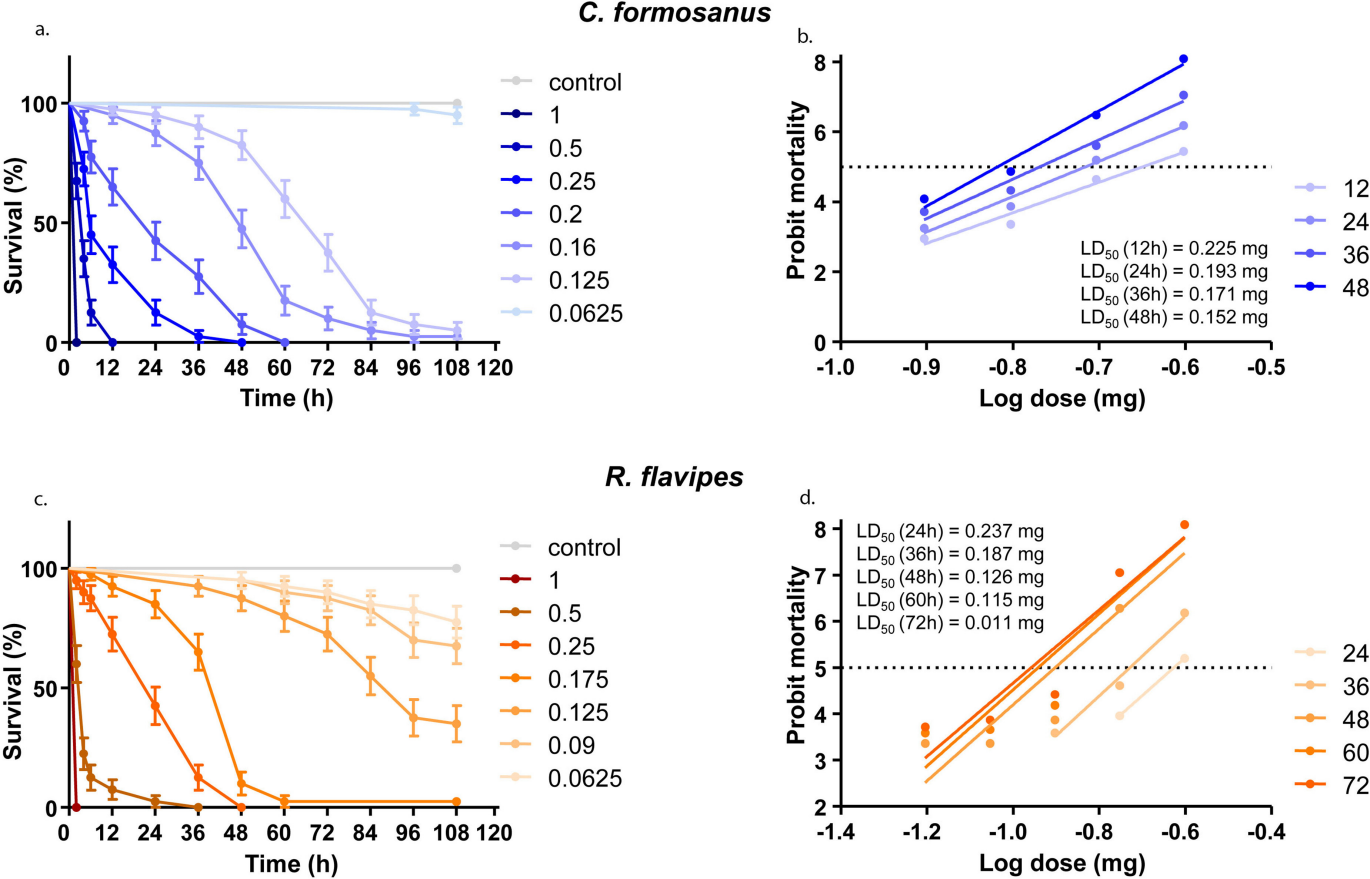
Survival curves for *C. formosanus* and *R. flavipes* upon eugenol treatment. **(a)** Survival curve for *C. formosanus* and seven eugenol doses. **(b)** Probit regression of *C. formosanus* survival data showing the calculated LD50 values for four defined intervals. **(c)** Survival curve for *R. flavipes* and seven eugenol doses. **(d)** Probit regression of *R. flavipes* survival data showing the calculated LD50 values for five defined intervals.

Termite survival in the mortality experiment showed a dynamic response to the gradient of eugenol doses applied, ranging from no mortality in the controls over 120 h to 100% mortality within 12 h at the highest dose of 1 mg (Figures 1a,c). The gradient of used doses allowed us to establish LD50 values for different time intervals (Figures 1b,d), from which we selected a 48 h experimental period as suitable for further downstream experiments. For this period, we established the sublethal dose of 0.0625 mg for both species, and the LD50 dose of 0.152 mg and 0.126 mg of eugenol for *C. formosanus* and *R. flavipes*, respectively. These doses were then used in the subsequent rearing of workers to gather samples for amplicon sequencing.

### 3.2 Sequencing statistics

Illumina paired-end sequencing of eugenol-treated (lethal and sublethal doses) and untreated (control) termite samples of two subterranean termites, *C. formosanus* and *R. flavipes*, yielded a total of 2,146,262 bacterial reads (Supplementary Excel 1). Following quality control with a Phred Quality score > 30, we obtained 1,851,435 clean reads for further bioinformatic analysis (Supplementary Excel 1).

### 3.3 Bacterial community structure

The bacterial sequences obtained from two subterranean termites were clustered into 2400 amplicon sequence variants (ASVs) at a 100% similarity level, which were subsequently annotated to 170 bacterial genera belonging to 21 phyla (Supplementary Excel 2). The Goods’ coverage (100%) and rarefaction curve illustrated the completeness of the sequencing, suggesting that most of the bacterial communities associated with all termite samples were covered during the sequencing (Supplementary Figure 1 and Supplementary Table 1).

#### 3.3.1 Comparative analysis of bacterial association in the two subterranean termites

**FIGURE 2:**
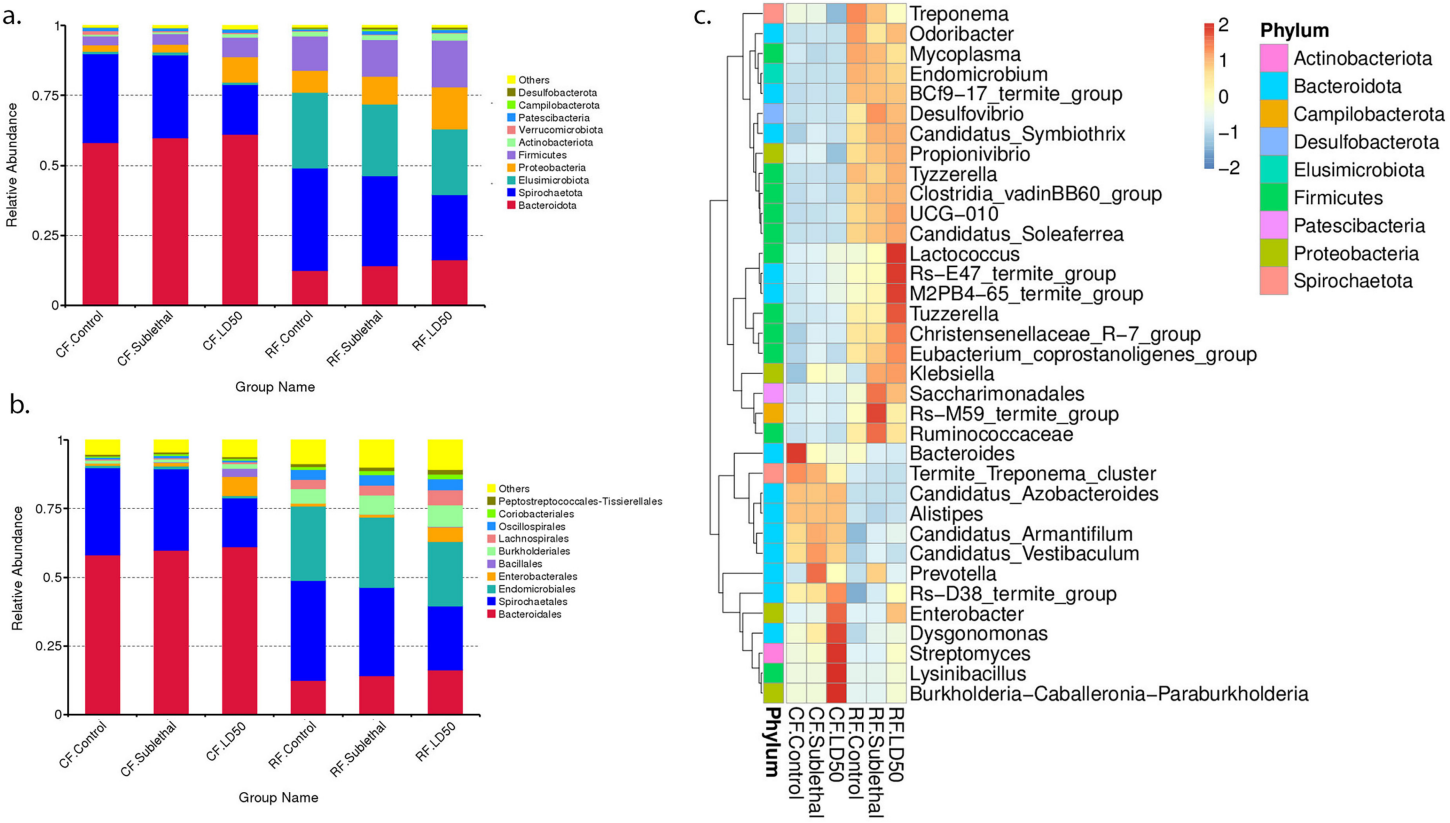
Bacterial diversity in two subterranean termites with and without eugenol treatment. The bar plot represents the relative abundance of bacterial communities (top 10) at the **(a)** phylum and **(b)** order level **(c)** Heatmap depicting the relative abundance of 35 dominant bacterial genera in the termite samples. The relative ASV abundance is represented by a color gradient where the darker color indicates higher abundance, whereas the lighter color indicates low abundance for a specific bacterial genus. CF- *C. formosanus*; RF- *R. flavipes*; LD50- lethal dose of eugenol.

**FIGURE 3:**
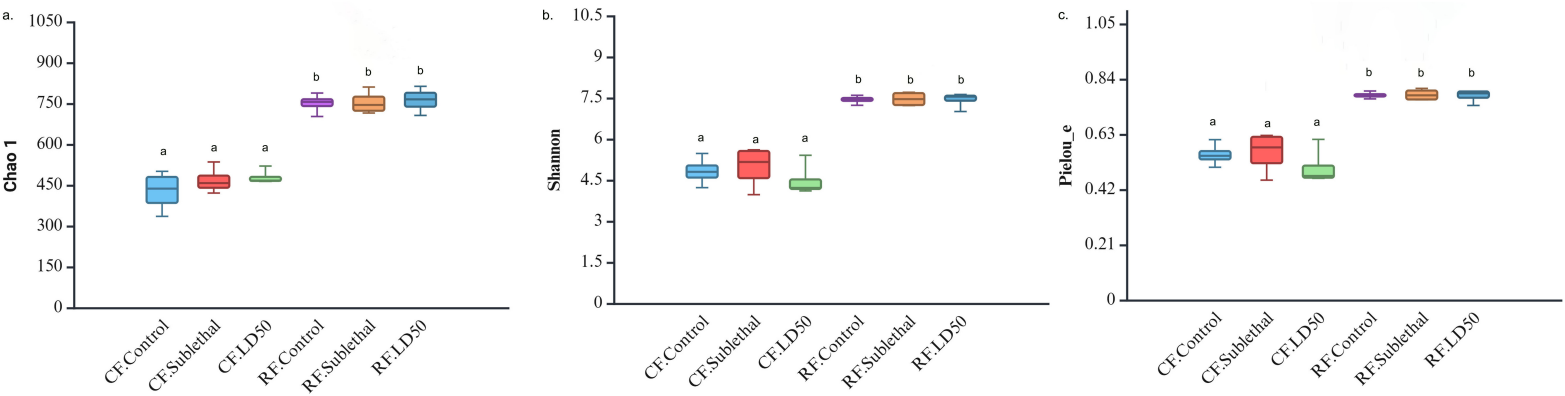
Boxplots depicting alpha diversity indices between different termite samples. **(a)** Bacterial richness estimated using the Chao1 estimation **(b)** Bacterial diversity illustrated by Shannon index **(c)** Bacterial evenness represented by Pielou index, indicating significant differences between the two subterranean termite species (*C. formosanus* and *R. flavipes*). Kruskal-Wallis pairwise group test was performed to statistically represent significant differences among different groups. Different letters denote statistical significance (*p* < 0.05). CF, *C. formosanus*; RF, *R. flavipes*; SL, sublethal dose of eugenol; LD50, lethal dose of eugenol.

The two subterranean lower termites (*C. formosanus* and *R. flavipes*) documented the presence of six dominant phyla, including Bacteroidota (CF.Control-58%; RF.Control-12%), Spirochaetota (CF.Control-31%; RF.Control-36%), Firmicutes (CF.Control-3.3%; RF.Control-12%), Proteobacteria (CF.Control-2%; RF.Control-8%), Actinobacteriota (CF.Control-0.6%; RF.Control-1%), and Elusimicrobiota (CF.Control-0.7%; RF.Control-27%) (Figure 2a and Supplementary Excel 2). The relative abundance of Bacteroidota was considerably higher in control *C. formosanus* (CF.Control- 58%), while Elusimicrobiota was highly prevalent in *R. flavipes* (RF.Control-27%) (Figure 2a and Supplementary Excel 2). Among the most abundant bacterial orders, Bacteroidales (CF.Control-58.2%, RF.Control-12.3%) were prevalent in CF.Control while Endomicrobiales (CF.Control-0.7%, RF.Control-27%), Lachnospirales (CF.Control-0.3%, RF.Control-3.3%), and Burkholderiales (CF.Control-0.9%, RF.Control-5.1%) were dominant in RF.Control (Figure 2b). The abundant bacterial genera include *Candidatus_Azobacteroides* (CF.Control 47%, RF.Control-1.9%), *Candidatus_Armanitifilum* (CF.Control 3%, RF.Control-1%), Termite_Treponema_cluster (CF.Control 8%, RF.Control-0.9%), Bacteroides (CF.Control 0.7%, RF.Control-0.2%), *Alistipes* (CF.Control 0.6%, RF.Control-0.1%) and *Candidatus_Vestibaculum* (CF.Control 2.5%, RF.Control-0.8%) that were dominant in CF.Control, whereas *Treponema* (CF.Control 19%, RF.Control-34%), *Endomicrobium* (CF.Control 0.7%, RF.Control-27%), Tuzzerella (CF.Control 0.09%, RF.Control-2%), *Candidatus_Symbiothrix* (CF.Control 0.4%, RF.Control-2%), *Odoribacter* (CF.Control 0.4%, RF.Control-1.3%), *Desulfovibrio* (CF.Control 0.06%, RF.Control-0.3%) *Tyzzerella* (CF.Control 0.01%, RF.Control-0.09%), *Ruminococcus* (CF.Control 0.02%, RF.Control-0.1%) and *Mycoplasma* (CF.Control 0.4%, RF.Control-1.3%) were prevalent in RF.Control (Figure 2c and Supplementary Excel 2). The alpha-diversity analyses revealed significantly higher bacterial richness (Chao1 index), diversity (Shannon index), and evenness (Pielou index) in *Reticulitermes* (RF.Control, Chao1 752.65 ± 17.87, Shannon index 7.458 ± 0.076, Pielou index 0.781 ± 0.008) compared to *Coptotermes* control samples (CF.Control, Chao1 429.82 ± 37.26, Shannon index 4.848 ± 0.258, Pielou index 0.555 ± 0.03) (*p* < 0.05) (Figure 3 and Supplementary Table 1).

**FIGURE 4:**
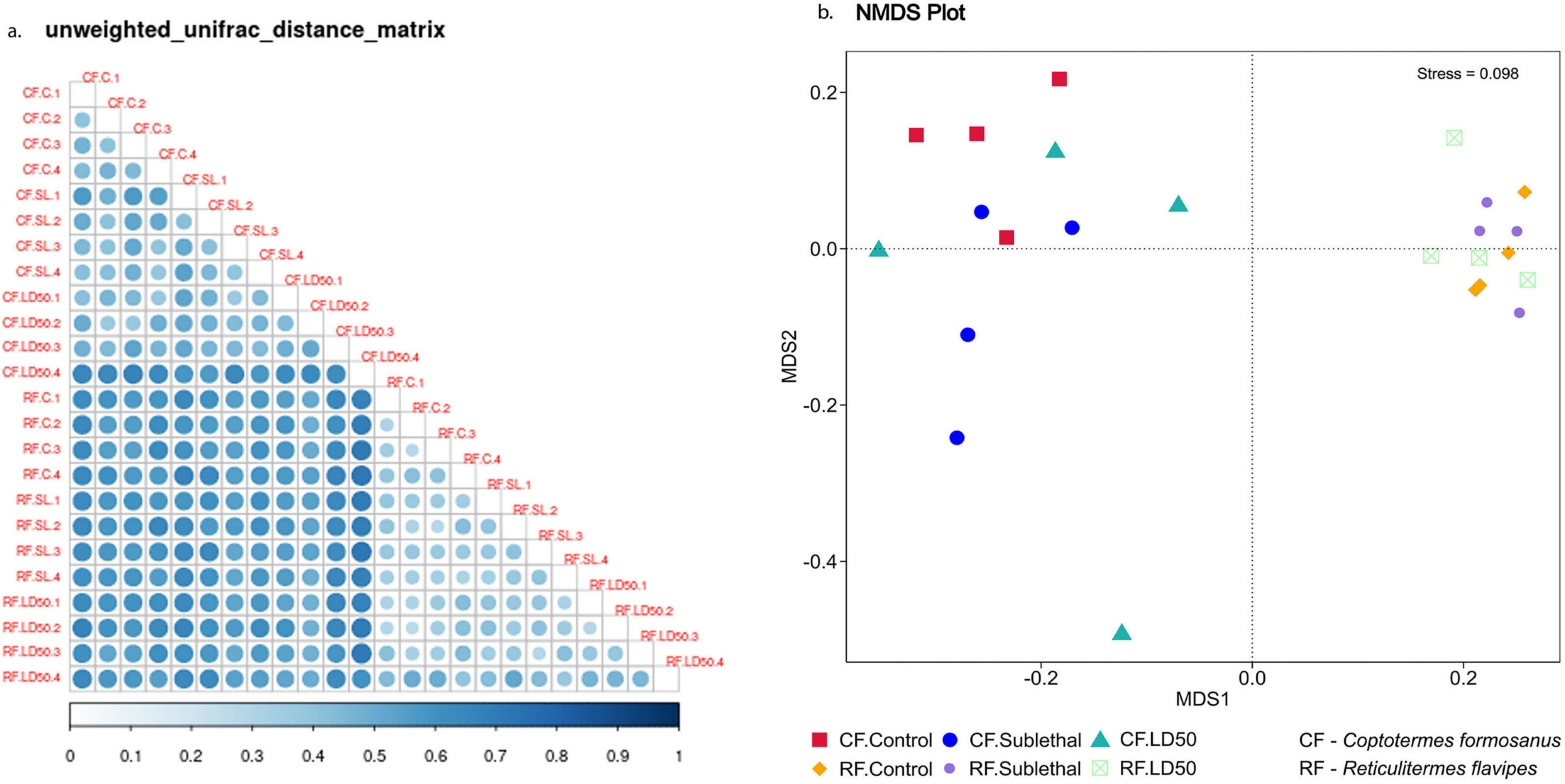
**(a)** The correlation matrix with unweighted UniFrac distances depicting the variation between the samples. **(b)** Non-metric Multi-Dimensional Scaling (NMDS) based on unweighted UniFrac distance matrix represents bacterial diversity variation in different termite samples. CF, *C. formosanus*; RF, *R. flavipes*; LD50, lethal dose of eugenol.

The unweighted UniFrac distance matrix illustrated the degree of correlation between the samples (Figure 4a). Beta diversity analysis based on unweighted UniFrac distances, as visualized in the NMDS plot, revealed two distinct clusters corresponding to the two lower termite species, indicating considerable differences in their overall bacterial community composition (Figure 4b). Additionally, ADONIS analysis revealed significant differences in the overall bacterial composition among the termite samples (Supplementary Table 2). Comparative analysis of two termite species revealed the presence of 308 shared ASVs, with 71 and 328 unique ASVs in CF.Control and RF.Control, respectively (Supplementary Figure 2a). Shared common bacterial ASVs were assigned to 12 phyla and 50 identified genera, including *Candidatus Azobacteroides, Endomicrobium, Tuzzerella, Treponema, Enterobacter, Termite Treponema cluster, Candidatus Armantifilum, Dysgonomonas, Rs-E47 termite group*, and *Candidatus Vestibaculum* (Supplementary Excel 3). Additionally, LEfSe analysis depicting the biomarkers documented *Candidatus Azobacteroides* and Termite *Treponema* cluster, members of *Dysgonomonadaceae* family, and Bacteroidota phylum as significant biomarkers for CF.Control (Supplementary Figure 2b). Conversely, members belonging to the family *Spirochaetaceae (Treponema)*, *Endomicrobiaceae (Endomicrobium)*, *Lachnospiraceae, Rhodocyclaceae*, Oscillospirales (order), Burkholderiales (order), and Clostridia (class) were identified as biomarkers in RF.Control (Supplementary Figure 2b). Furthermore, MetaStat and *t*-test analyses indicated differentially abundant bacterial genera, including *Raoultibacter*, *Alistipes*, *Candidatus Azobacteroides, Termite Treponema cluster, Candidatus Armantifilum, Candidatus Vestibaculum*, and *Dysgonomonas*, that were significantly higher in CF.Control, whereas *Treponema, Endomicrobium, Candidatus Symbiothrix, Tuzzerella, Ruminococcus*, *Margulisbacteria*, and *Mycoplasma* were abundant in RF.Control (Supplementary Figure 3 and Supplementary Tables 3, 4).

#### 3.3.2 Impact of eugenol exposure on the *Coptotermes formosanus* bacterial assemblages

Eugenol treatment (sublethal and lethal doses) altered the bacterial abundances in *C. formosanus.* At lethal concentration (LD50 doses of eugenol), Spirochaetota abundance was reduced (CF.LD50, 17.8%) compared to untreated control (CF.Control-31.8%). In contrast, Bacteroidota (CF.LD50-61%, CF.Sublethal-60% CF.Control-58%), Firmicutes (CF.LD50-6.9%, CF.Sublethal-3.7%, CF.Control-3.3%), Proteobacteria (CF.LD50-9.1%, CF.Sublethal-2.7%, CF.Control-2.2%) and Actinobacteriota (CF.LD50-1.4%, CF.Sublethal-0.8%, CF.Control-0.6%), showed increased relative abundances on eugenol treatment compared to control (Figure 2a and Supplementary Excel 2). Furthermore, eugenol treatment increased the relative abundances of bacterial genera, including *Burkholderia–Caballeronia-Paraburkholderia*, *Lysinibacillus*, *Streptomyces*, *Dysgonomonas*, *Candidatus Armantifilum, Candidatus Vestibaculum, Enterbacter, Prevotella*, and Rs-D38_termite_group (Figure 2c and Supplementary Excel 2). The alpha diversity analysis indicated no significant differences in the bacterial richness, diversity, and evenness among the eugenol-treated (sublethal or LD50 doses) termite samples and the untreated *C. formosanus* control termites (Figure 3 and Supplementary Table 1). Similarly, PCoA analysis revealing the beta diversity among the bacterial population in the *C. formosanus* samples indicated no distinct clustering, suggesting that the overall bacterial diversity in *C. formosanus* samples on eugenol treatment did not differ substantially (Supplementary Figure 4a). However, there were differences in the relative abundance of the bacterial communities.

**FIGURE 5:**
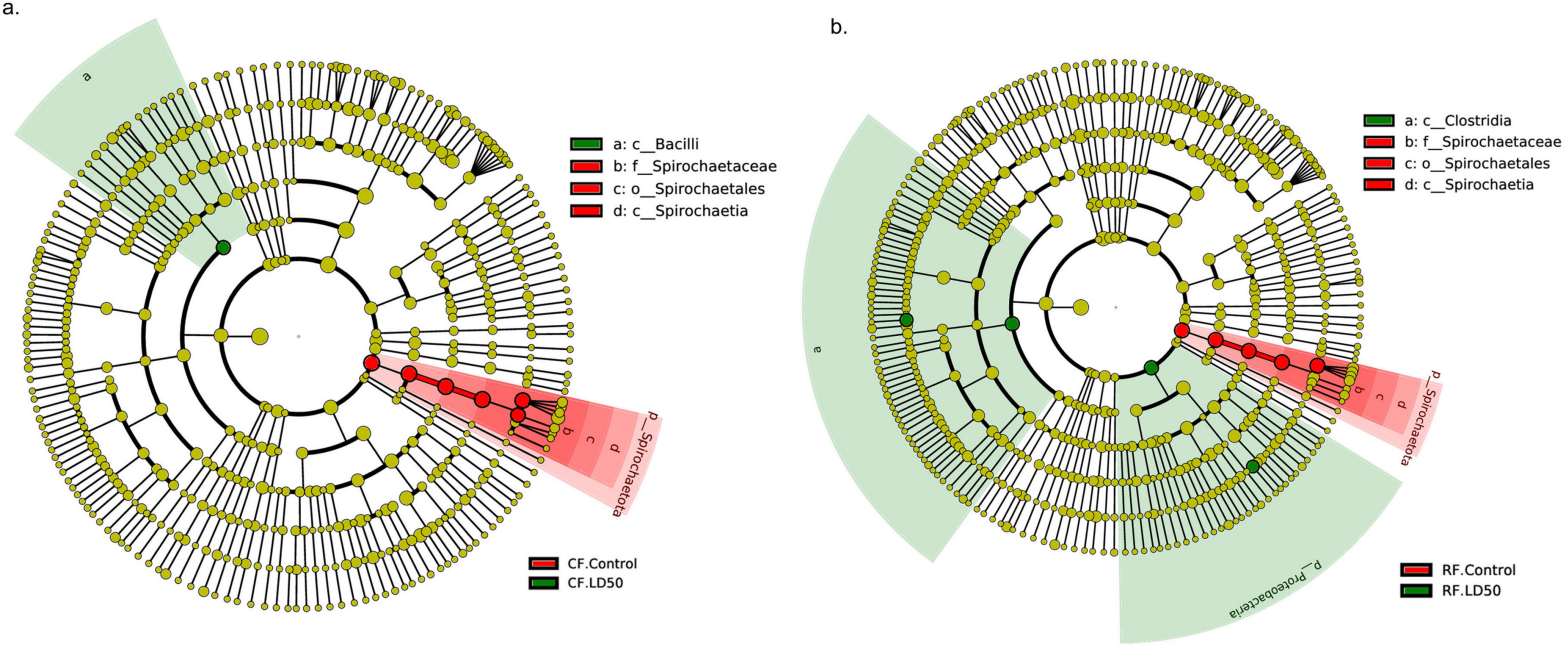
LefSe analysis. Cladogram representing significant bacterial biomarkers among **(a)** Eugenol-treated *C. formosanus* and the control termite. **(b)** Eugenol-treated *R. flavipes* and the control termite. The distinct taxonomic level (phylum to genus) is denoted in the circle from inward to outward. The different colored nodes (red, green, blue, and purple) represent bacterial species that play significant roles in termites. CF, *C. formosanus*; RF, *R. flavipes*.

The differentially abundant bacterial communities identified by *t*-test and MetaStat analysis showed significantly higher abundances of *Candidatus Symbiothrix, Pilibacter*, and Ruminococcaceae in CF.Sublethal compared to CF.Control (Supplementary Tables 3, 4). In CF.LD50, *Dysgonomonas, Tuzzerella, Rs-E47 termite group, Pilibacter*, and *Lactococcus* were significantly enriched, while *Treponema, Termite Treponema cluster, Fretibacterium, Spirochaeta*, and *Candidatus Ancillula* showed significantly reduced abundance (Supplementary Tables 3, 4). LEfSe analysis highlighted Spirochaetota (including *Treponema* and *Termite Treponema cluster*) as predominant in CF.Control, whereas bacterial genus belonging to class *Bacilli* was dominant in CF.LD50 (Figure 5a and Supplementary Figure 5a).

#### 3.3.3 Eugenol exposure on the *Reticulitermes flavipes* bacterial assemblages

Similar to the *C. formosanus* bacteriome, eugenol treatment to *R. flavipes* showed a decline in the relative abundance of the bacterial phyla, including Spirochaetota (RF.LD50-23.4%, RF.Sublethal-32.2%, RF.Control-36.6%), Elusimicrobiota (RF.LD50-23.4%, RF.Sublethal-25.6%, RF.Control- 7%) compared to the untreated control termite. On the contrary, Firmicutes (RF.LD50- 16.6%, RF.Sublethal-13.08%, RF.Control- 12.2%), Bacteoidota (RF.LD50- 16.2%, RF.Sublethal-14.1%, RF.Control-12.4%) Proteobacteria (RF.LD50- 5.1%, RF.Sublethal-9.84%, RF.Control-8%), and Actinobacteriota (RF.LD50-2.52%, RF.Sublethal-1.74%, RF.Control-1.63%) documented increased abundance on eugenol treatment (Figure 2a and Supplementary Excel 2). Additionally, the bacterial genera including *Lactococcus*, *Eubacterium*, *Tuzzerella*, *Desulfovibrio*, *Candidatus_Soleaferra*, *Klebsiella*, and members of Saccharimonadales, *Rs-M59_termite_group*, Ruminococcaceae showed increased abundance on eugenol treatment, while *Treponema*, *Mycoplasma*, and *Endomicrobium* showed reduced abundance (Figure 2c). Our results revealed no significant differences in bacterial richness, diversity, and evenness in *R. flavipes* following eugenol treatment (Figure 3 and Supplementary Table 1). Moreover, the overall bacterial diversity illustrated by the PCoA plot showed no distinct clustering of the termite samples (with and without eugenol treatment), suggesting no drastic impact of the eugenol treatment on the termite bacterial assemblages (Supplementary Figure 4b).

*T*-test and MetaStat analysis revealed that *Tuzzerella, Enterobacter, Rs-E47 termite group, Lactococcus, Dysgonomonas*, Saccharimonadales, and *Pilibacter* were significantly abundant in RF.LD50 samples, while Saccharimonadales*, Desulfovibrio, Lactococcus*, and *Candidatus Saccharimonas* were significantly abundant in RF.Sublethal (Supplementary Tables 3, 4). However, eugenol treatment significantly reduced *Treponema* (Supplementary Excel 2). LEfSe analysis indicated that Spirochaetota members, including *Treponema*, were dominant in RF.Control, whereas RF.LD50 samples exhibited enrichment in Proteobacteria (e.g., *Enterobacter*) and Firmicutes (e.g., *Tuzzerella*) (Figure 5b and Supplementary Figure 5b). Our results suggest that eugenol exposure does not significantly affect the overall bacterial communities in the two subterranean termite species. However, there is a considerable reduction in the relative abundance of Spirochaetota and an increased abundance of Firmicutes and Proteobacteria on treatment with eugenol (LD50 doses) in both *C. formosanus* and *R. flavipes*.

### 3.4 Quantitative PCR (qPCR) assay

**FIGURE 6:**
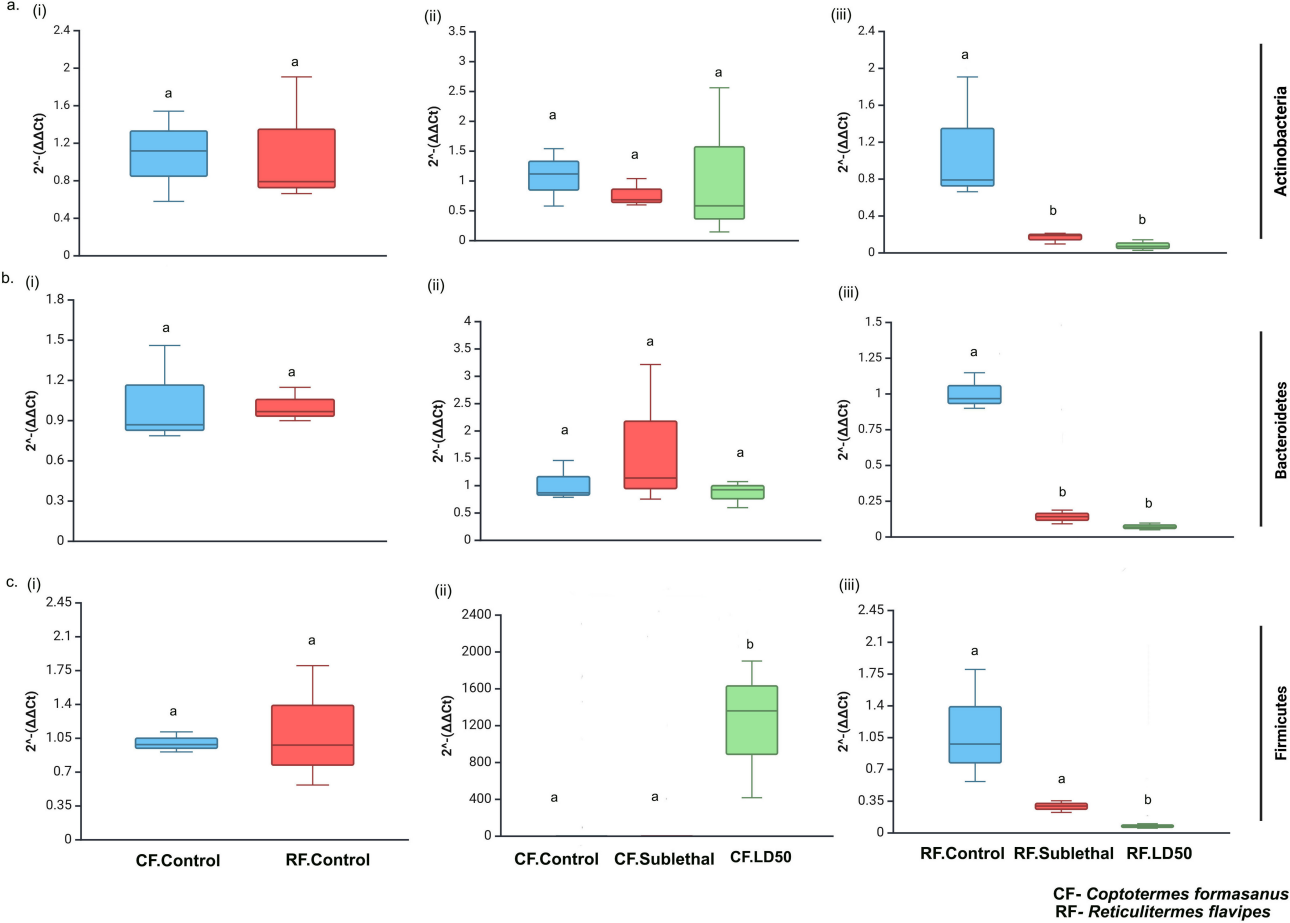
Real-time qPCR assay. Box plot represents the relative abundance of selected bacterial taxa **(a)** Actinobacteria, **(b)** Bacteroidetes, and **(c)** Firmicutes present in two termite species under different conditions, such as (i) between two subterranean termite species, (ii) eugenol-treated and untreated *C. formosanus* samples, and (iii) eugenol-treated and untreated *R. flavipes* samples. The 2^∧^(-ΔΔCt) revealed the fold change of the bacterial abundance relative to the stable reference gene (EF1alpha). The data indicate the mean fold change in bacterial abundance within the samples (*n* = 3). The statistical comparison was performed using the one-way ANOVA with Tukey multiple comparisons test. Different letters denote statistical significance (*p* < 0.05). CF, *C. formosanus*; RF, *R. flavipes*; LD50, lethal dose of eugenol.

The qPCR analysis targeting selected bacterial phyla (Actinobacteria, Bacteroidetes, and Firmicutes) demonstrated that the relative fold change in Actinobacteria, Bacteroidetes, and Firmicutes abundance did not differ significantly between the two termite species (Figure 6). Within species, eugenol-treated *C. formosanus* did not show a significant difference in Actinobacteria and Bacteroidetes abundance compared with its untreated control (Figures 6a,b). In contrast, Firmicutes abundance was significantly higher in LD50 eugenol-treated *C. formosanus* than in the untreated control (Figure 6c). For *R. flavipes*, the untreated control showed significantly higher abundance of Actinobacteria, Bacteroidetes, and Firmicutes than LD50 eugenol-treated samples (Figures 6a–c). Comparison of overall bacterial load revealed a significant difference in total bacterial abundance between the two subterranean termite species (Supplementary Figure 6a). However, no significant difference in overall bacterial abundance was observed between the eugenol-treated and untreated controls within each species (Supplementary Figures 6b,c). It is important to note that the qPCR results were only partly consistent with the sequencing-based abundance patterns. In *C. formosanus*, the increased abundance of Firmicutes under LD50 eugenol treatment was consistent with the amplicon data. However, in *R. flavipes*, qPCR showed reduced abundance of Actinobacteria, Bacteroidetes, and Firmicutes in LD50-treated samples relative to controls, contrasting with the sequencing-based relative abundance trends. This discrepancy may reflect methodological differences between amplicon-based relative profiling and qPCR-based taxon-specific quantification.

### 3.5 Putative functional role of termite bacteriome

**FIGURE 7:**
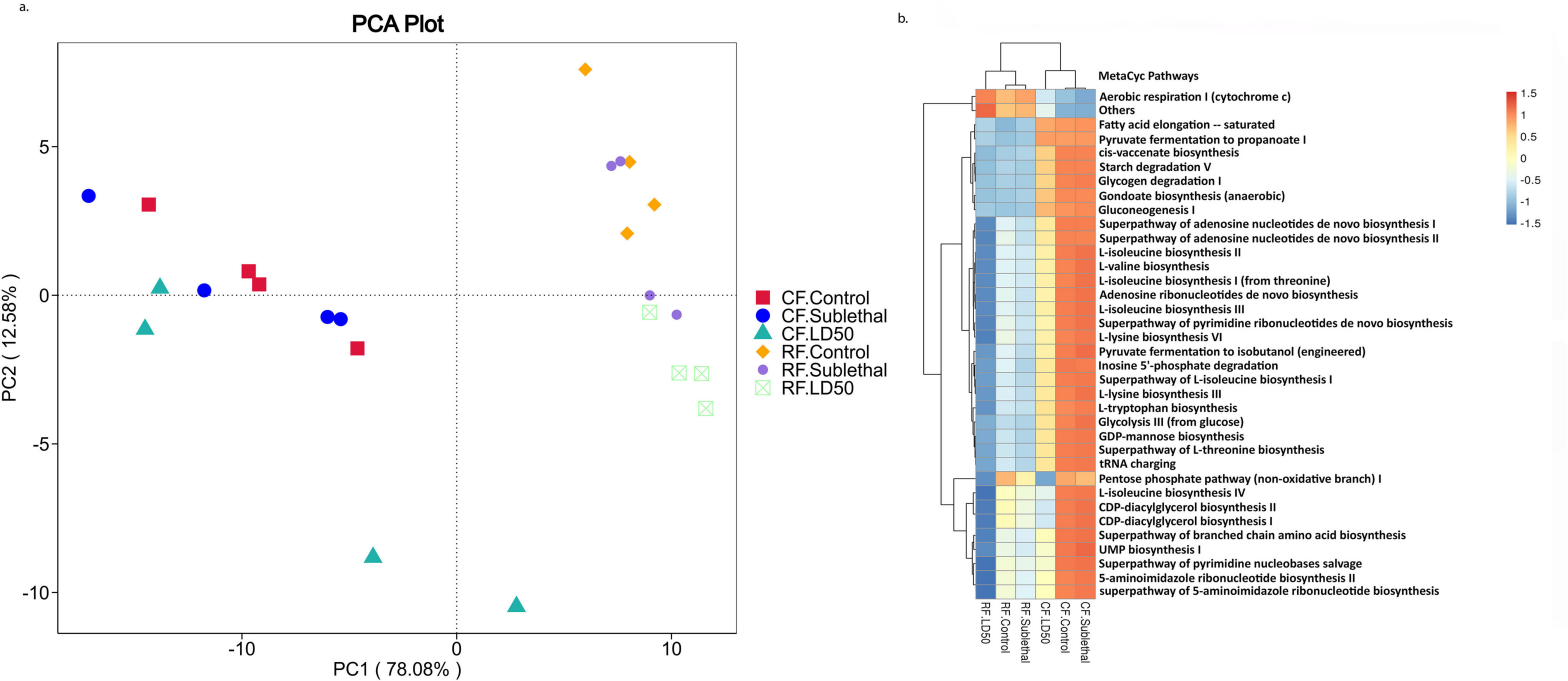
PICRUSt2 analysis. **(a)** The PCA plot illustrates distinct clusters of predicted pathways between the two termite species, suggesting variation in the abundance of bacterial pathways predicted by PICRUSt2 software. **(b)** Heatmap showing the differences in the abundance of the top 35 bacterial pathways predicted in termite samples with or without eugenol treatment based on the Metacyc database. The color gradient indicates the relative abundance of each predicted MetaCyc pathway, where the darker color indicates higher abundance, whereas the lighter color indicates low abundance. CF, *C. formosanus*; RF, *R. flavipes*; LD50, lethal dose of eugenol.

PICRUSt2 analysis documented the putative functional potential of the bacterial community in termite samples based on the contributions of each ASV to specific functions annotated in different databases (Figure 7 and Supplementary Figure 7). Predicted functional profiles differed primarily between termite species. The metabolic pathways between the two lower termites were grouped into two distinct clusters, suggesting that the abundance and diversity of the bacterial consortium differed between the two termite species and that the pathways showed variation (Figure 7a). The heatmaps based on different databases predicted functional differences within the two lower termites on eugenol treatments (Figure 7b and Supplementary Figure 7).

PICRUSt2 prediction software revealed the presence of 6179 KEGG orthologs (KO database) and 1949 enzymes (EC database), categorized into 381 MetaCyc pathways (MetaCyc database) (Supplementary Excel 4). The most abundant KOs and enzymes were correlated with the MetaCyc pathways, including metabolism (carbohydrate, amino acid, nucleic acid, and fatty acid), transporters (sugars, proteins, lipoproteins, toxic compounds, and ions), vitamin biosynthesis, DNA replication, repair and recombination, transcription, transcription and translation factors, and bacterial motility proteins, among others were predominant across the control and eugenol-treated samples of both the termite species (Supplementary Table 5 and Supplementary Excel 4). Notably, MetaCyc pathways, KOs, and ECs revealed a lower relative abundance of carbohydrate degradation, glycolysis, pentose phosphate pathway, pyruvate fermentation, amino acid, fatty acid, and nucleic acid biosynthesis pathways in LD50 samples compared to the controls of both termites (Figure 7 and Supplementary Figure 7). Toxic compound extrusion-related membrane proteins (DinF/NorM/MATE family Na + -driven multidrug efflux pump, ABC-type multidrug transport system, membrane protein involved in the export of O-antigen and teichoic acid, outer membrane protein OmpA, multidrug efflux pump subunit AcrA, and Outer membrane protein TolC) and a MarR family DNA-binding transcriptional regulator predicted from the COG database were present in higher abundance in LD50 dose eugenol-treated samples of both termites (Supplementary Figure 7c). Furthermore, the schematic diagram depicts the enzymes involved in the wood degradation pathway that were predicted by PICRUSt2 (Supplementary Figure 8). The abundance of most of the enzymes involved in wood degradation was marginally reduced on LD50 eugenol-treated termite samples (Supplementary Excel 4). As PICRUSt2 provides only predictive insights into the putative functional potential of microbial communities, these findings necessitate further validation through metatranscriptomic analyses and complementary functional assays.

## 4 Discussion

The symbiotic relationship between termites and their microbial associates is fundamental to termite survival and evolutionary success. Beyond their nutritional role, these symbionts serve diverse physiological and ecological functions (Hongoh, 2011; Peterson and Scharf, 2016b; Arora et al., 2022). In lower termites, the symbiosis is predominantly focused on protists, which are critical for the digestion of lignocellulosic material (Brune, 2014; Peterson and Scharf, 2016a); however, bacterial symbionts also play indispensable roles in carbon and nitrogen metabolism. Despite this, comparative metagenomic insights into the bacterial communities of economically important subterranean termites, *C. formosanus* and *R. flavipes*, remain limited (Dar et al., 2022; Chakraborty et al., 2023b). The present study provides the first comparative analysis of the bacterial communities associated with these two termite species and examines how sublethal and lethal eugenol exposure reshapes their bacterial assemblages.

Although the subterranean termites, *C. formosanus* (native to East Asia) and *R. flavipes* (native to North America), occupy similar ecological niches, they represent phylogenetically distinct lineages (Rust and Su, 2012; Bourguignon et al., 2014). Our results demonstrate that host evolutionary divergence is reflected in distinct bacterial community structures. Both species shared a conserved core bacteriome dominated by Bacteroidota, Spirochaetota, Elusimicrobiota, Firmicutes, Proteobacteria, and Actinobacteria; however, relative abundances of these groups vary significantly. Such interspecific differences in microbial composition can be attributed to a variety of factors, including host phylogenetic divergence (Tai et al., 2015), vertical inheritance of microbes and host-microbe co-evolution (Abdul Rahman et al., 2015; Groussin et al., 2020), ecological habitat differentiation (Yun et al., 2014), dietary preferences (Mikaelyan et al., 2015), and underlying host genetic backgrounds (Greenwood et al., 2021).

Interestingly, a shared hallmark of both termite species is the dominance of Spirochaetota, particularly the genus *Treponema*, an observation consistently reported in prior studies (Su et al., 2016; Zeng et al., 2021; Chakraborty et al., 2023b). Although *Treponema* was abundant in both species, *R. flavipes* demonstrated a notably higher relative abundance. The multifaceted functions of Spirochaetota, including *Treponema*, in degrading polysaccharides, acetogenesis, and nitrogen fixation as termite gut symbionts, make them crucial for the fitness and survival of the termites (Leadbetter et al., 1999; Lilburn et al., 2001; Graber and Breznak, 2004; Dröge et al., 2008; Ohkuma et al., 2015; Tokuda et al., 2018). In *C. formosanus*, Bacteroidetes were relatively more abundant, particularly *Candidatus Azobacteroides*, which has been identified as a key diazotroph involved in nitrogen fixation and the biosynthesis of amino acids and cofactors (Noda et al., 2005; Hongoh et al., 2008), whereas *R. flavipes* harbored greater proportions of Elusimicrobiota, especially *Endomicrobium*, known as a specific endosymbiont of termite gut protists with critical roles in amino acid and cofactor biosynthesis (Stingl et al., 2005; Ikeda-Ohtsubo and Brune, 2009; Brune, 2012; Brune, 2014). Other abundant members, including *Candidatus Vestibaculum* and *Candidatus Armantifolium*, were reported to aid in nitrogen fixation and supply nitrogenous nutrients for the host protists (Stingl et al., 2004; Ohkuma, 2008; Desai and Brune, 2012). Conversely, *R. flavipes* showed a predominance of *Candidatus Symbiothrix*, a genus implicated in lignocellulose degradation and amino acid biosynthesis (Yuki et al., 2015). Since bacterial communities belonging to the family Bacteroidales can ferment polysaccharides and produce cellulases and other fiber-degrading enzymes, these epibionts might compensate for host enzyme deficiencies (Stingl et al., 2004). Furthermore, the higher relative abundance of Firmicutes in *R. flavipes* likely enhances its capabilities for carbohydrate degradation, fermentation, nitrogen fixation, and acetogenesis (Husseneder, 2010; Brune, 2014). In parallel, genera within Proteobacteria, notably *Enterobacter*, *Acinetobacter*, and *Propionivibrio*, were detected in both species and are known for their roles in lignocellulose breakdown, acetate fermentation, and nitrogen fixation (Potrikus and Breznak, 1977; Brune et al., 2002; König, 2006; Kannisto et al., 2015). The differences in bacterial community composition may indicate that while both termite species depend on similar metabolic capabilities, the bacterial taxa responsible for performing these functions differ between the hosts. The coexistence of such functional overlap and taxonomic divergence may confer stability to the holobiont resilience by preserving essential metabolic processes while enabling host-specific ecological specialization. Nevertheless, further investigations are required to comprehend the role of these bacterial communities in the termite holobiont.

Eugenol, a phenolic monoterpenoid found in several plants such as clove, basil, etc., is known for its well-documented broad-spectrum insecticidal and antibacterial properties (Cornelius et al., 1997; Huang et al., 2002; Xie et al., 2015; Elbestawy et al., 2023; Olaimat et al., 2024). However, despite these well-established properties, our findings revealed no significant alteration in overall bacterial richness or diversity in the *C. formosanus* and *R. flavipes* following exposure to eugenol. This suggests that while eugenol possesses potent antibacterial activity, its impact on the termite bacterial communities may not manifest as a broad-spectrum disruption of microbial diversity. Instead, eugenol appears to exert selective pressures on specific bacterial taxa, as evidenced by significant shifts in the relative abundance of key microbial groups. Most notably, treatment with eugenol at LD50 concentrations resulted in a decline in the abundance of Spirochaetota, a dominant and functionally essential phylum in both termite species. Genera such as *Treponema* (in both species) and the *Termite Treponema cluster* (in *C. formosanus*) were significantly depleted following eugenol exposure. The selective reduction of Spirochaetota under eugenol exposure may be partly due to the lipophilic phenolic structure of eugenol that disrupts bacterial membranes increasing permeability and interfering with proton gradients and enzyme activity (Gill and Holley, 2006). Spirochaetota, including members of the genus *Treponema*, possess a distinctive diderm cell envelope with a relatively flexible outer membrane and periplasmic flagella (Zückert, 2019), features that are thought to support motility and specialized metabolic functions within the termite gut (Graber and Breznak, 2004; Limberger, 2004; Ohkuma et al., 2015). However, this structural organization may also render them more susceptible to membrane-active compounds such as eugenol, which can compromise membrane integrity and disrupt energy metabolism. Alterations in the abundance Spirochaetota might potentially affect essential processes like lignocellulose degradation, acetogenesis, nitrogen fixation, and nitrogen recycling, which are crucial for fulfilling the carbon, nitrogen, and energy requirements necessary for termite health and vitality (Graber and Breznak, 2004; Dröge et al., 2008; Hongoh, 2011; Ohkuma et al., 2015; Lin et al., 2022). Previous studies have demonstrated that the reduction of Spirochaetota significantly impairs termite survival (Eutick et al., 1978), further highlighting the ecological relevance of these symbionts. In addition to Spirochaetota, we observed a reduction in Bacteroides abundance in both termite species, which may compromise carbohydrate degradation and fermentation (Sakamoto and Ohkuma, 2013). In *C. formosanus*, eugenol treatment significantly reduced *Candidatus Ancilulla*, a symbiont predicted to contribute to sugar metabolism and the biosynthesis of amino acids, vitamins, and cofactors (Strassert et al., 2016). These declines highlight the potential of eugenol to disrupt critical metabolic pathways, thereby jeopardizing termite nutritional homeostasis.

In contrast to these reductions, specific microbial taxa increased in abundance following eugenol exposure. Notably, members of the phylum Firmicutes, including *Pilibacter*, *Lactococcus*, and *Tuzzerella*, were significantly enriched in both termite species. The enrichment of these taxa may reflect a selective advantage under chemical stress, as these genera are functionally associated with carbohydrate degradation, acetate production, and nitrogen fixation (Husseneder, 2010). Specifically, two lactic acid bacteria *Pilibacter* and *Lactococcus*, are implicated in sugar metabolism and lactic acid fermentation, processes that help stabilize gut pH and preserve microbial homeostasis (Hutkins and Nannen, 1993; Higashiguchi et al., 2006; Noda et al., 2018). The proliferation of such taxa under eugenol exposure may therefore represent a compensatory response aimed at maintaining gut functionality despite disrupting other microbial groups. Furthermore, *Lactococcus* is known for its capacity to synthesize vitamins, contributing to host nutrition (Li et al., 2023). This suggests that eugenol-induced shifts in bacterial composition not only reflect selective tolerance but may also reinforce symbiotic functions to support termite survival under chemical stress.

Considerable shifts were also observed in the Proteobacteria community after eugenol treatment. In *C. formosanus*, there was a prominent increase in the *Burkholderia–Caballeronia–Paraburkholderia* complex, potentially linked to detoxification and nutrient cycling (Kaltenpoth and Flórez, 2020). Similarly, *Enterobacter* and *Klebsiella* were prevalent in *R. flavipes* on eugenol exposure, which might be attributed to lignocellulose digestion and nitrogen fixation (Behar et al., 2005; König, 2006; Doolittle et al., 2008; Korsa et al., 2022). Notably, these genera are known to harbor multidrug efflux pumps and detoxification systems that may facilitate resistance to antimicrobial compounds like eugenol (Confer and Ayalew, 2013; Abdali et al., 2017). Their proliferation, therefore, suggests that bacterial communities may undergo functional realignment to enhance chemical tolerance while sustaining key metabolic processes. Additionally, genera such as *Dysgonomonas* and *Prevotella*, both associated with carbohydrate degradation and fermentation, were significantly abundant in eugenol-treated termites (Pramono et al., 2015; Bridges and Gage, 2021; Dao et al., 2021; Shinkai et al., 2022). The increased abundance of these fermentative taxa may represent a compensatory adjustment, ensuring continued energy production and maintaining gut metabolic balance despite disruption of the native community structure. These shifts indicate that bacterial taxa capable of stress resistance and metabolic redundancy are selectively favored under eugenol challenge.

Interestingly, the endosymbiotic bacteria *Candidatus Azobacteroides* in *C. formosanus* and *Endomicrobium* in *R. flavipes*, which reside within gut protists, did not exhibit significant changes in abundance following eugenol exposure. This may be linked to a combination of factors, including lower sensitivity to eugenol, detoxification mechanisms, or physical protection within protist hosts. Despite observable disruptions to free-living bacteria, the apparent resilience of these protist-associated endosymbionts features the complexity of the termite holobiont. It also supports the hypothesis that while protists remain stable, bacterial symbionts are central to mediating physiological responses to environmental stressors (Peterson et al., 2015). However, conducting more extensive and rigorous research to validate this hypothesis is crucial.

The core bacteriome in two lower termites included members from bacterial phyla Bacteroidota, Firmicutes, Spirochaetota, and Proteobacteria. This core bacterial consortium is well documented to perform crucial metabolic functions, including acetogenesis, N_2_ fixation, sugar degradation and fermentation, and biosynthesis of amino acids, vitamins, and cofactors in subterranean termites (Husseneder, 2010). Notably, while exposure to eugenol did not alter the overall composition of this core bacteriome, it induced significant shifts in the relative abundance of several phyla and their constituent genera in both termite species. This observation suggests that eugenol may not disrupt bacterial diversity at the community level but instead selectively modulates the abundance of key microbial taxa. For instance, eugenol treatment reduced the abundance of *Treponema*, a core member of the gut bacteriome, in both subterranean termite species. Given the established role of *Treponema* in essential processes such as lignocellulose degradation, acetogenesis, and nitrogen recycling, perturbations to its population can have direct consequences on termite nutrition and overall colony function (Eutick et al., 1978). Such taxon-specific perturbations underline the ecological impact of eugenol, which targets critical microbial functions without eliminating overall diversity. Consequently, eugenol can be understood as a selective disruptor of termite symbioses, with implications for termite ecology and the strategic development of pest management approaches that exploit vulnerabilities in the termite holobiont.

PICRUSt2-based functional predictions indicated that several metabolic pathways declined following lethal (LD50) eugenol exposure, whereas sublethal doses were associated with their relative enrichment. This pattern suggests that sublethal exposure may trigger compensatory upregulation of microbial functions related to detoxification and stress adaptation, while LD50 levels exceed the microbial threshold for resilience, leading to reduced functional capacity. Enrichment of enzymes and pathways, such as β-glucosidase, acetolactate synthase, pyruvate fermentation (PWY-5100), and the pentose phosphate pathway, under sublethal conditions, suggests a potential enhancement of lignocellulose degradation (Supplementary Figure 7). Although these predictions provide valuable insight, they represent inferred functional potential rather than measured activity. Hence, experimental validation using metatranscriptomics, metaproteomics, metabolomics, or enzyme assays is needed to confirm these findings.

Beyond functional inference, these results hold applied significance. Disruption of termite-associated bacterial assemblages compromises key physiological processes, including cellulose digestion, immune defense, and reproduction, thereby undermining colony fitness (Rosengaus et al., 2011; Peterson et al., 2015; Tang et al., 2023). In this context, the ability of eugenol to selectively modulate bacterial composition and function highlights its potential as an eco-friendly biocontrol agent that weakens termites through targeted symbiont disruption (Rupawate et al., 2023). Despite its potential, the practical implementation of eugenol under field conditions remains constrained by its inherent physicochemical instability. High volatility and susceptibility to oxidative degradation can diminish environmental persistence and reduce eugenol’s bioactive concentrations (Woranuch and Yoksan, 2013). To overcome these limitations, advanced formulation approaches, particularly micro- and nano-encapsulation, offer practical solutions (Ayllón-Gutiérrez et al., 2024). Encapsulation enhances molecular stability, protects eugenol from premature oxidation and volatilization, and facilitates controlled or sustained release. Collectively, these strategies improve insecticidal performance, extend residual activity, and enhance functional durability under variable environmental conditions (Ayllón-Gutiérrez et al., 2024; Yammine et al., 2024). Beyond its direct application, eugenol also serves as a model phytochemical exemplifying how small aromatic xenobiotics can exert selective pressure on specific microbial taxa, thereby reshaping community structure and modulating metabolic functions.

## 5 Limitations

While this study provides new insights into termite-associated bacterial dynamics under eugenol exposure, several limitations should be considered when interpreting the findings. The study relies on bacterial 16S rRNA amplicon sequencing and PICRUSt2-based functional prediction, which provide only inferred functional potential rather than direct evidence of microbial activity, and thus require validation through approaches such as metatranscriptomics, metaproteomics, or targeted biochemical assays. The observed shifts in the bacterial community composition under eugenol exposure cannot be attributed solely to direct antimicrobial effects, as host-mediated stress responses may also play a significant role. Eugenol may exert its toxic effects by disrupting the termite holobiont as a whole, affecting both host physiology and the associated gut microbial community, with the combined disturbance of these interdependent components potentially contributing to termite mortality. Future studies integrating *in vitro* antibacterial assays, measurements of host physiological responses, and functional validation approaches will be needed to distinguish the relative roles of direct antimicrobial effects and host-mediated gut dysbiosis in contributing to the observed toxicity. Furthermore, the experimental design is limited to a single time point (48 h) and two dose levels (sublethal and LD50), restricting insights into temporal dynamics and dose–response relationships. Inclusion of intermediate eugenol concentrations and later sampling intervals (e.g., 72 and 96 h) would be valuable in future work to further define the long-term dose-response relationship and progression of flora and functional changes following eugenol exposure. Additionally, the study focuses exclusively on bacterial communities and does not account for gut protozoa, which are central to lignocellulose digestion in the lower termites. The absence of protozoan data, therefore, imposes some limits on our understanding of termite holobiont responses to eugenol. The qPCR validation was limited to a few phylum-level bacterial groups and did not target lineage-specific taxa characteristic of the termite gut bacteriome. Finally, the laboratory-based filter-paper assay may not fully reflect natural termite foraging conditions. In natural environments, eugenol would be encountered within complex wood matrices, where its stability, bioavailability, and interactions with microbial communities may differ substantially. Therefore, caution should be exercised when extrapolating these findings to field conditions or practical pest management applications. Addressing these limitations through integrative, multi-omics approaches, expanded experimental designs, and ecologically relevant exposure systems will be essential for advancing mechanistic understanding and evaluating the applied potential of microbiome-targeted termite control strategies. Nevertheless, our current findings pinpoint the delicate interdependence between termites and their bacterial assemblages and provide a foundational framework for future research aimed at elucidating how phytochemicals influence termite-associated microbial communities.

## 6 Conclusion

The present study delivers a comparative analysis of the bacterial communities associated with two subterranean termites, *C. formosanus* and *R. flavipes*, revealing significant interspecific differences likely shaped by evolutionary divergence, continental origins, vertical transmission of symbionts, and host genetic background. Exposure to eugenol was associated with significant termite mortality and taxon-specific shifts in bacterial community composition without altering overall bacterial diversity, emphasizing its capacity to modulate termite symbioses without collapsing community structure. However, the extent to which these bacteriome changes arise from direct antimicrobial effects of eugenol versus indirect host-mediated responses remains unresolved. The resulting toxicity and termite mortality are likely not attributable solely to bacterial community disruption but may arise from a combined effect involving perturbation of the gut microbiota (both bacterial and fungal) and changes in termite gene expression. Future integration of metatranscriptomics, metabolomics, and functional validation will be critical to unravel the underlying mechanisms, assess ecological significance, and evaluate their utility for sustainable termite management.

## Data Availability

The datasets presented in this study can be found in online repositories. The names of the repository/repositories and accession number(s) can be found in the article/Supplementary material.

## Footnotes

1
http://ccb.jhu.edu/software/FLASH/

2
http://www.arb-silva.de/

3
http://huttenhower.sph.harvard.edu/lefse/

## Supplementary material

Supplementary Figure 1 Rarefaction curves. Different colors and symbols indicate different samples. CF, *C. formosanus*; RF, *R. flavipes*; LD50, lethal dose of eugenol.

Supplementary Figure 2 **(a)** Venn diagram showing the bacterial ASVs contribution between the two termites and the abundant core bacterial communities shared between the two termite species. **(b)** Cladogram representing significant bacterial biomarkers between the two termite species. Distinct taxonomic level (phylum to genus) is denoted in the circle from inward to outward. The different colored nodes (red, green, blue and purple) represent bacterial species that play a significant role in the termites. CF, *C. formosanus*; RF, *R. flavipes*; LD50, lethal dose of eugenol.

Supplementary Figure 3 *T*-test analysis illustrating significant bacterial communities in two untreated lower termite species. CF, *C. formosanus*; RF, *R. flavipes*.

Supplementary Figure 4 PCoA analysis. **(a)** PCoA plot representing the beta diversity based on Bray-Curtis distance in eugenol-treated and untreated control *C. formosanus* samples. **(b)** PCoA plot representing the beta diversity based on Bray-Curtis distance in eugenol-treated and untreated control *R. flavipes* samples. LD50, lethal dose of eugenol.

Supplementary Figure 5 LEfSe analysis. Histogram representing the LDA scores depicting the significantly abundant bacterial communities. **(a)** Eugenol-treated and untreated *C. formosanus* samples, **(b)** eugenol-treated *R. flavipes*, and the untreated termite control. CF- *C. formosanus*; RF- *R. flavipes*; LD50- lethal dose of eugenol.

Supplementary Figure 6 qPCR assay. Bacterial 16S rRNA gene copy numbers in *C. formosanus* and *R. flavipes* in different conditions, such as (i) between two subterranean termite species, (ii) eugenol-treated and untreated *C. formosanus* samples, and (iii) eugenol-treated and untreated *R. flavipes* samples. Data was log-transformed for better visualization. The statistical comparison was performed using the one-way ANOVA with Tukey multiple comparisons test.

Supplementary Figure 7 Functional prediction using PICRUSt2. Heatmap illustrates the putative bacterial functional contribution based on **(a)** KEGG, **(b)** EC, and **(c)** COG databases.

Supplementary Figure 8 Schematic diagram of the enzymes predicted using PICRUSt 2 that are involved in the wood degradation pathway.

Supplementary Excel 1 Raw and clean bacterial reads.

Supplementary Excel 2 ASV table indicating the relative abundance of bacterial communities in two termite species (with or without eugenol treatment).

Supplementary Excel 3 Common and Unique bacteriome in two subterranean termite species.

Supplementary Excel 4 PICRUSt2 analysis indicating the relative abundance of the predicted functions in the termite samples based on the KEGG, EC, COG, and Metacyc databases.
