# Supplementary material for: Subterranean synergies: termite bacterial diversity and eugenol-mediated selective dysbiosis
Source: Front Microbiol. 2026 Jun 17;17:1818254. doi: 10.3389/fmicb.2026.1818254 (PMC13318894; doi:10.3389/fmicb.2026.1818254)
Supplement: Supplementary file 1 [file Data_Sheet_1.pdf]

Supplementary Tables

Supplementary Table 1: Alpha diversity indices. The data represented the mean value ±SE for four biological replicates across different termite samples. (SE-Standard Error).CF- *Coptotermes formosanus*; RF- *Reticulitermes flavipes*; SL- sub-lethal dose of eugenol; LD50- lethal dose of eugenol.

Sample	Good's Coverage (%)	Chao1	Shannon Index	Pielou Index
CF.Control	100	429.82±52.69	4.848±0.364	0.555±0.03
CF.Sublethal	100	469.99±34.59	4.997±0.544	0.562±0.056
CF.LD50	100	481.51±19.28	4.508±0.435	0.506±0.050
RF.Control	100	752.65±25.27	7.458±0.107	0.781±0.008
RF.Sublethal	100	755.83±30.46	7.483±0.185	0.783±0.015
RF.LD50	100	764.56±32.34	7.455±0.203	0.778±0.017

Supplementary Table 2: ADONIS analysis evaluating the significant difference between the overall bacterial diversity within the two subterranean termites (Df = degree of freedom, SS = sums of squares of deviations, MS = SS/Df, F. Model = F-test value, R2 = the ratio of grouping variance and total variance). Values in parentheses illustrate Residual Error. The p-value represents the significant variation in bacterial diversity between the two termite species.

Analysis	Df	SS	MS	F.Model	R2	Pr(>F)
16S	5	1.4353	0.2871	3.0135	0.4557	0.001

Supplementary Table 3: Metastat analysis representing the top 10 differentially abundant bacterial genera across different termite samples. The FDR test evaluates the significance of observed abundance differences among the samples. CF- *Coptotermes formosanus*; RF- *Reticulitermes flavipes*; SL- sub-lethal dose of eugenol; LD50- lethal dose of eugenol.

Sample comparisons	Samples	Significantly higher bacterial genera [P<0.05]
**Species-specific microbial association (CF.C vs RF.C)**		
**CF.C vs RF.C**	CF.C	*Candidatus Azobacteroides*, Termite *Treponema* cluster, *Candidatus Armantifilum*, *Candidatus Vestibaculum*, *Dysgonomonas*
	RF.C	*Treponema, Endomicrobium*, *Candidatus Symbiothrix*, *Tuzzerella*, *Mycoplasma*
**Impact of eugenol on microbial association in *Coptotermes formosanus* (CF.C vs CF.SL; CF.C vs CF.LD50)**		
**CF.C vs CF.SL**	CF.C	*Pseudoxanthomonas*
	CF.SL	*Candidatus Symbiothrix*, *Pilibacter*, *Ruminococcaceae*
**CF.C vs CF.LD50**	CF.C	*Treponema*, Termite_*Treponema*_cluster, *Fretibacterium*, *Spirochaeta*, *Candidatus Ancillula*
	CF.LD50	*Dysgonomonas*, *Tuzzerella*, Rs-E47_termite_group, *Pilibacter*, *Lactococcus*
**Impact of eugenol on microbial association in *Reticulitermes flavipes* (RF.C vs RF.SL; RF.C vs RF.LD50)**		
**RF.C vs RF.SL**	RF.C	*Candidatus Ancillula*, A21b, *Raoultibacter*, *Margulisbacteria*
	RF.SL	*Saccharimonadales*, *Desulfovibrio*, *Lactococcus*, *Candidatus Saccharimonas*
**RF.C vs RF.LD50**	RF.C	*Treponema*
	RF.LD50	*Tuzzerella*, *Enterobacter,* Rs-E47_termite_group, *Lactococcus*, *Dysgonomonas*, *Saccharimonadales*, *Pilibacter*

Supplementary Table 4: *t*-test analysis illustrating significant bacterial communities in different termite samples. CF- *Coptotermes formosanus*; RF- *Reticulitermes flavipes*; SL- sub-lethal dose of eugenol; LD50- lethal dose of eugenol

Sample comparisons	Samples	Significantly higher bacterial genera [P<0.05]
**Species-specific microbial association (CF.C vs RF.C)**		
**CF.C vs RF.C**	CF.C	*Candidatus Azobacteroides*, Termite *Treponema* cluster, *Candidatus Armantifilum*, *Candidatus Vestibaculum*, *Alistipes*
	RF.C	*Treponema, Endomicrobium*, *Candidatus Symbiothrix*, *Tuzzerella*, *Mycoplasma*
**Impact of eugenol on microbial association in *Coptotermes formosanus* (CF.C vs CF.SL; CF.C vs CF.LD50)**		
**CF.C vs CF.SL**	CF.C	-
	CF.SL	*Pilibacter*
**CF.C vs CF.LD50**	CF.C	*Treponema*, Termite_*Treponema*_cluster
	CF.LD50	*Pilibacter*
**Impact of eugenol on microbial association in *Reticulitermes flavipes* (RF.C vs RF.SL; RF.C vs RF.LD50)**		
**RF.C vs RF.SL**	RF.C	*Raoultibacter*
	RF.SL	-
**RF.C vs RF.LD50**	RF.C	-
	RF.LD50	***Tuzzerella***, Rs-E47_termite_group, ***Saccharimonadales***, *Lactovum*, ***Pilibacter***

Supplementary Table 5: Description table for predicted top (a) KEGG Orthologs, (b) Enzymes, and (c) MetaCyc Pathways, and their putative functions

**KEGG Orthologs**		
**Entry**	**Name**	**Function**
K00266	Glutamate synthase (NADPH) small chain [EC:1.4.1.13]	Amino acid biosynthesis, L-glutamate Biosynthesis I
K03406	Methyl-accepting chemotaxis protein	Bacterial motility proteins
K01190	Beta-galactosidase [EC:3.2.1.23]	Carbohydrate Degradation
K05349	Beta-glucosidase [EC:3.2.1.21]	Carbohydrate Degradation
K06142	Outer membrane protein	DNA condensation
K03655	ATP-dependent DNA helicase RecG [EC:5.6.2.4]	DNA repair and recombination
K03654	ATP-dependent DNA helicase RecQ [EC:5.6.2.4]	DNA repair and recombination
K03530	DNA-binding protein HU-beta	DNA repair and recombination
K03701	Excinuclease ABC subunit A	DNA repair and recombination
K02342	DNA polymerase III subunit epsilon [EC:2.7.7.7]	DNA replication
K00059	3-oxoacyl-[acyl-carrier protein] reductase [EC:1.1.1.100]	Fatty acid biosynthesis
K01897	Long-chain acyl-CoA synthetase [EC:6.2.1.3]	Fatty acid biosynthesis
K02428	XTP/dITP diphosphohydrolase [EC:3.6.1.66]	Nucleotide metabolism
K00615	Transketolase [EC:2.2.1.1]	Pentose Phosphate Pathways
K03797	Carboxyl-terminal processing protease [EC:3.4.21.102]	Post-translational modification
K07056	16S rRNA (cytidine1402-2'-O)-methyltransferase [EC:2.1.1.198]	Ribosome biogenesis
K06180	23S rRNA pseudouridine1911/1915/1917 synthase [EC:5.4.99.23]	Ribosome biogenesis
K02529	LacI family transcriptional regulator	Transcription factors
K03086	RNA polymerase primary sigma factor	Transcription machinery
K03088	RNA polymerase sigma-70 factor, ECF subfamily	Transcription machinery
K02355	Elongation factor G	Translation factors
K01990	ABC-2 type transport system ATP-binding protein	Transporters
K01992	ABC-2 type transport system permease protein	Transporters
K06147	ATP-binding cassette, subfamily B, bacterial	Transporters
K07114	Ca-activated chloride channel homolog	Transporters
K02005	HlyD family secretion protein	Transporters
K02003	Putative ABC transport system ATP-binding protein	Transporters

K02004	Putative ABC transport system permease protein	Transporters
K09808	Lipoprotein-releasing system permease protein	Transporters, lipoprotein transport
K02014	Iron complex outermembrane receptor	Transporters, protein transport
K02057	Simple sugar transport system permease protein	Transporters, sugar transport
K00791	tRNA dimethylallyltransferase [EC:2.5.1.75]	tRNA biogenesis
K07133	Uncharacterized protein	-
K06889	Uncharacterized protein	-
K07090	Uncharacterized protein	-
**Enzyme Nomenclature**		
**EC Number**	**Enazyme Name**	**Function**
EC 1.6.5.3	NADH:ubiquinone reductase (H(+)-translocating)	Aerobic respiration I (cytochrome c)
EC 4.2.1.33	3-isopropylmalate dehydratase	Amino acid biosynthesis, Superpathway of branched chain amino acid biosynthesis, L-leucine Biosymthesis
EC 1.4.1.14	Glutamate synthase (NADH)	Amino acid biosynthesis, L-glutamate Biosynthesis I
EC 1.4.1.13	Glutamate synthase (NADPH)	Amino acid biosynthesis, L-glutamate Biosynthesis I
EC 4.2.1.35	(R)-2-methylmalate dehydratase	Amino acid biosynthesis, L-isoleucine biosynthesis II
EC 4.2.1.20	Tryptophan synthase	Amino acid biosynthesis, L-tryptophan biosynthesis
EC 2.2.1.6	Acetolactate synthase	Amino acid biosynthesis, Superpathway of branched chain amino acid biosynthesis
EC 6.3.5.6	Asparaginyl-tRNA synthase (glutamine-hydrolyzing)	Aminoacyl-tRNA Charging
EC 6.3.5.7	Glutaminyl-tRNA synthase (glutamine-hydrolyzing)	Aminoacyl-tRNA Charging
EC 6.1.1.20	Phenylalanine--tRNA ligase	Aminoacyl-tRNA charging
EC 3.2.1.23	Beta-galactosidase	Carbohydrate Degradation
EC 3.2.1.21	Beta-glucosidase	Carbohydrate Degradation
EC 1.97.1.4	[Formate-C-acetyltransferase]-activating enzyme	Carbohydrate metabolism
EC 3.2.1.52	Beta-N-acetylhexosaminidase	Carbohydrate metabolism
EC 3.6.4.12	DNA helicase	DNA repair and recombination
EC 5.99.1.3	DNA topoisomerase (ATP-hydrolyzing)	DNA replication
EC 2.7.7.7	DNA-directed DNA polymerase	DNA replication
EC 3.1.26.4	Ribonuclease H	DNA replication
EC 2.1.1.72	Site-specific DNA-methyltransferase (adenine-specific)	DNA restriction-modification systems

EC 3.1.21.3	Type I site-specific deoxyribonuclease	DNA restriction-modification systems
EC 6.4.1.2	Acetyl-CoA carboxylase	Fatty Acid Biosynthesis
EC 1.1.1.100	3-oxoacyl-[acyl-carrier-protein] reductase	Fatty acid biosynthesis
EC 6.2.1.3	Long-chain-fatty-acid--CoA ligase	Fatty acid biosynthesis
EC 5.4.2.12	Phosphoglycerate mutase (2, 3-diphosphoglycerate-independent)	Glycolysis
EC 1.8.98.1	Dihydromethanophenazine:CoB--CoM heterodisulfide reductase	Methanogenesis
EC 5.2.1.8	Peptidylprolyl isomerase	Protein folding
EC 3.4.21.102	C-terminal processing peptidase	Proteolysis
EC 2.7.2.1	Acetate kinase	Pyruvate fermentation to acetate and lactate II
EC 2.3.1.8	Phosphate acetyltransferase	Pyruvate fermentation to acetate and lactate II
EC 1.2.7.1	Pyruvate synthase	Pyruvate fermentation to acetate and lactate II
EC 5.4.99.23	23S rRNA pseudouridine(1911/1915/1917) synthase	Ribosome biogenesis
EC 1.2.7.11	2-oxoacid oxidoreductase (ferredoxin)	TCA cycle
EC 1.2.7.3	2-oxoglutarate synthase	TCA cycle
EC 2.7.7.6	DNA-directed RNA polymerase	Transcription
EC 2.7.13.3	Histidine kinase	Transferase
EC 3.6.3.14	H(+)-transporting two-sector ATPase	Transporters, ions
EC 2.7.1.69	Protein-N(pi)-phosphohistidine--D-mannose phosphotransferase	Transporters, sugar transport
EC 2.8.1.7	Cysteine desulfurase	Vitamin Biosynthesis
**MetaCyc Pathways**		
**BioCyc ID**	**Pathway**	**Function**
PWY-3781	Aerobic respiration I (cytochrome c)	Aerobic respiration I (cytochrome c)
ILEUSYN-PWY	L-isoleucine biosynthesis I (from threonine)	Amino-acid biosynthesis
PWY-5101	L-isoleucine biosynthesis II	Amino-acid biosynthesis
PWY-5103	L-isoleucine biosynthesis III	Amino-acid biosynthesis
PWY-5104	L-isoleucine biosynthesis IV	Amino-acid biosynthesis
PWY-2942	L-lysine biosynthesis III	Amino-acid biosynthesis
PWY-5097	L-lysine biosynthesis VI	Amino-acid biosynthesis
TRPSYN-PWY	L-tryptophan biosynthesis	Amino-acid biosynthesis
VALSYN-PWY	L-valine biosynthesis	Amino-acid biosynthesis
BRANCHED-CHAIN-AA-SYN-PWY	Superpathway of branched chain amino acid biosynthesis	Amino-acid biosynthesis
PWY-3001	Superpathway of L-isoleucine biosynthesis I	Amino-acid biosynthesis

THRESYN-PWY	Superpathway of L-threonine biosynthesis	Amino-acid biosynthesis
TRNA-CHARGING-PWY	tRNA charging	Aminoacyl- tRNA charging
PWY-5659	GDP-mannose biosynthesis	Carbohydrate Biosynthesis
GLUCONEO-PWY	Gluconeogenesis I	Carbohydrate Biosynthesis
GLYCOCAT-PWY	Glycogen degradation I	Carbohydrate Degradation
PWY-6737	Starch degradation V	Carbohydrate Degradation
PWY-5667	CDP-diacylglycerol biosynthesis I	Fatty Acid Biosynthesis
PWY0-1319	CDP-diacylglycerol biosynthesis II	Fatty Acid Biosynthesis
PWY-5973	cis-vaccenate biosynthesis	Fatty Acid Biosynthesis
FASYN-ELONG-PWY	Fatty acid elongation – saturated	Fatty Acid Biosynthesis
PWY-7663	Gondoate biosynthesis (anaerobic)	Fatty Acid Biosynthesis
ANAGLYCOLYSIS-PWY	Glycolysis III (from glucose)	Glycolysis
PWY-6122	5-aminoimidazole ribonucleotide biosynthesis II	Nucleoside and Nucleotide Biosynthesis
PWY-7219	Adenosine ribonucleotides de novo biosynthesis	Nucleoside and Nucleotide Biosynthesis
PWY-6277	Superpathway of 5-aminoimidazole ribonucleotide biosynthesis	Nucleoside and Nucleotide Biosynthesis
PWY-7229	Superpathway of adenosine nucleotides de novo biosynthesis I	Nucleoside and Nucleotide Biosynthesis
PWY-6126	Superpathway of adenosine nucleotides de novo biosynthesis II	Nucleoside and Nucleotide Biosynthesis
PWY-7208	Superpathway of pyrimidine nucleobases salvage	Nucleoside and Nucleotide Biosynthesis
PWY0-162	Superpathway of pyrimidine ribonucleotides de novo biosynthesis	Nucleoside and Nucleotide Biosynthesis
PWY-5686	UMP biosynthesis I	Nucleoside and Nucleotide Biosynthesis
PWY-5695	Inosine 5'-phosphate degradation	Nucleoside and Nucleotide Degradation
NONOXIPENT-PWY	Pentose phosphate pathway (non-oxidative branch) I	Pentose Phosphate Pathways
PWY-5100	Pyruvate fermentation to acetate and lactate II	Pyruvate Fermentation
PWY-7111	Pyruvate fermentation to isobutanol (engineered)	Pyruvate Fermentation
P108-PWY	Pyruvate fermentation to propanoate I	Pyruvate Fermentation
PWY-6969	TCA cycle V (2-oxoglutarate:ferredoxin oxidoreductase)	TCA cycle
