# Supplementary figures and images for: Subterranean synergies: termite bacterial diversity and eugenol-mediated selective dysbiosis
Source: Front Microbiol. 2026 Jun 17;17:1818254. doi: 10.3389/fmicb.2026.1818254 (PMC13318894; doi:10.3389/fmicb.2026.1818254)

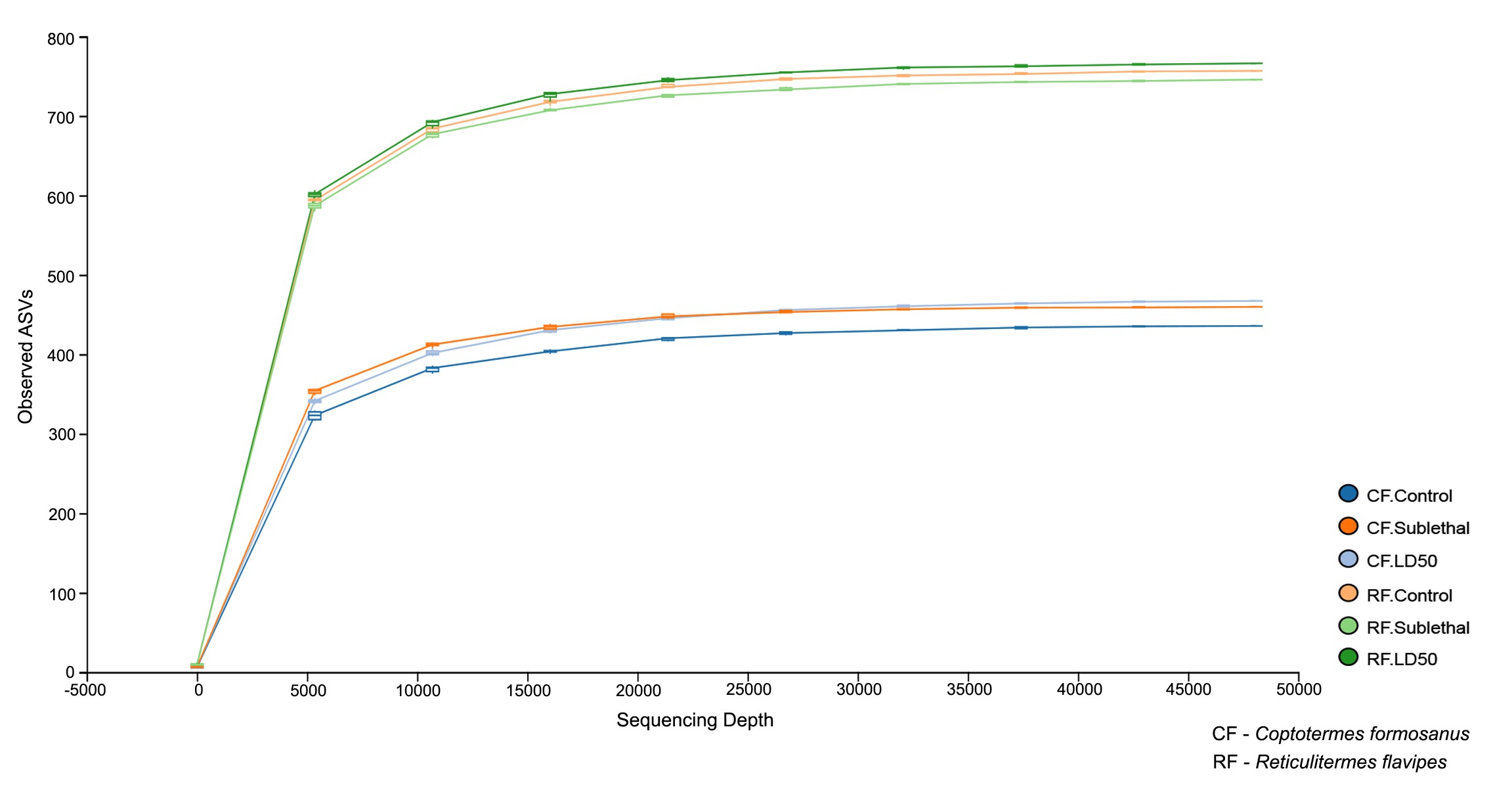

Supplement: Supplementary Figure 1 — Rarefaction curves. Different colors and symbols indicate different samples. CF, C. formosanus; RF, R. flavipes; LD50, lethal dose of eugenol. [file Image_1.jpeg]

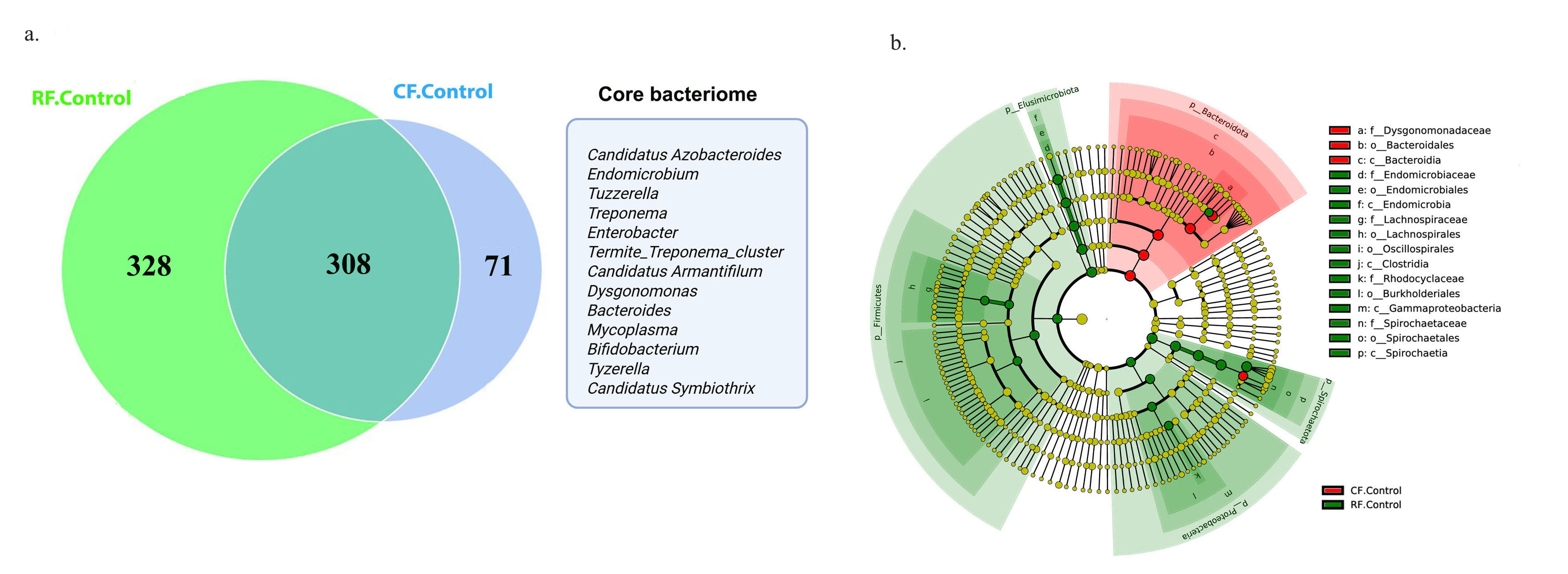

Supplement: Supplementary Figure 2 — (a) Venn diagram showing the bacterial ASVs contribution between the two termites and the abundant core bacterial communities shared between the two termite species. (b) Cladogram representing significant bacterial biomarkers between the two termite species. Distinct taxonomic level (phylum to genus) is denoted in the circle from inward to outward. The different colored nodes (red, green, blue and purple) represent bacterial species that play a significant role in the termites. CF, C. formosanus; RF, R. flavipes; LD50, lethal dose of eugenol. [file Image_2.jpeg]

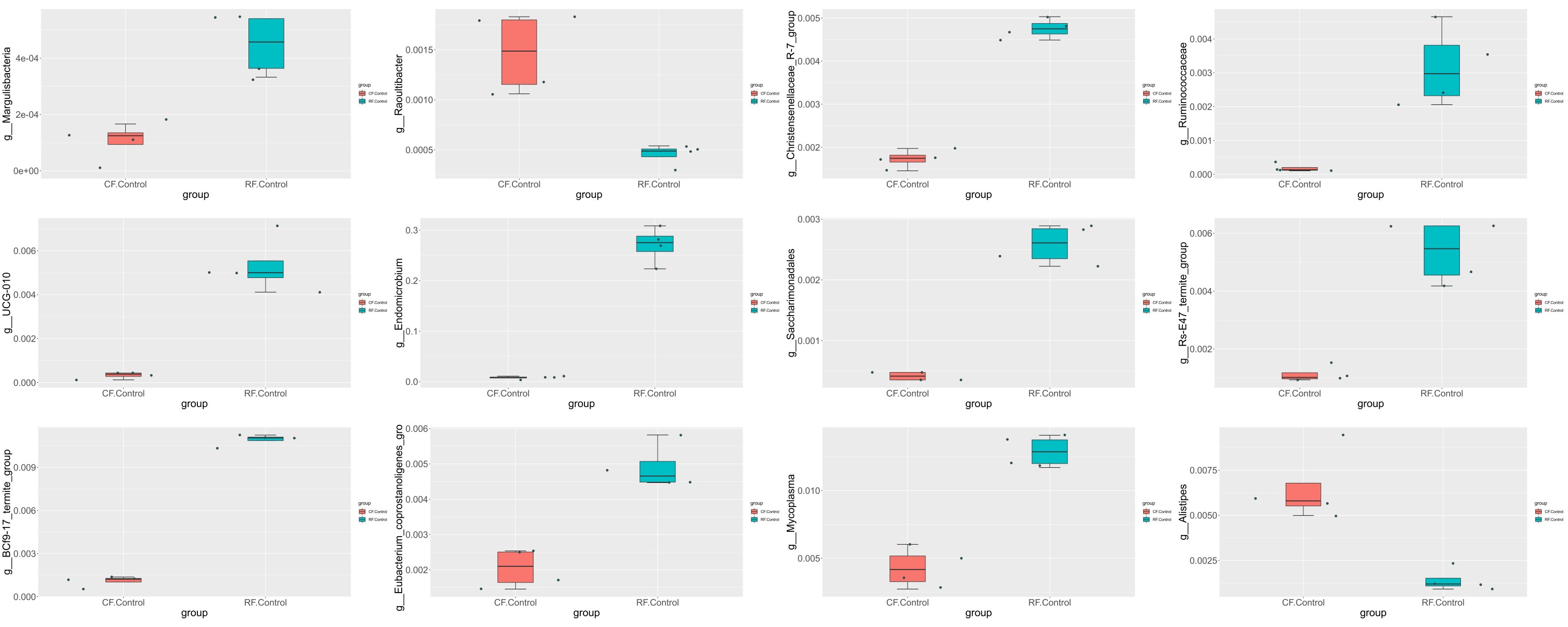

Supplement: Supplementary Figure 3 — T-test analysis illustrating significant bacterial communities in two untreated lower termite species. CF, C. formosanus; RF, R. flavipes. [file Image_3.jpeg]

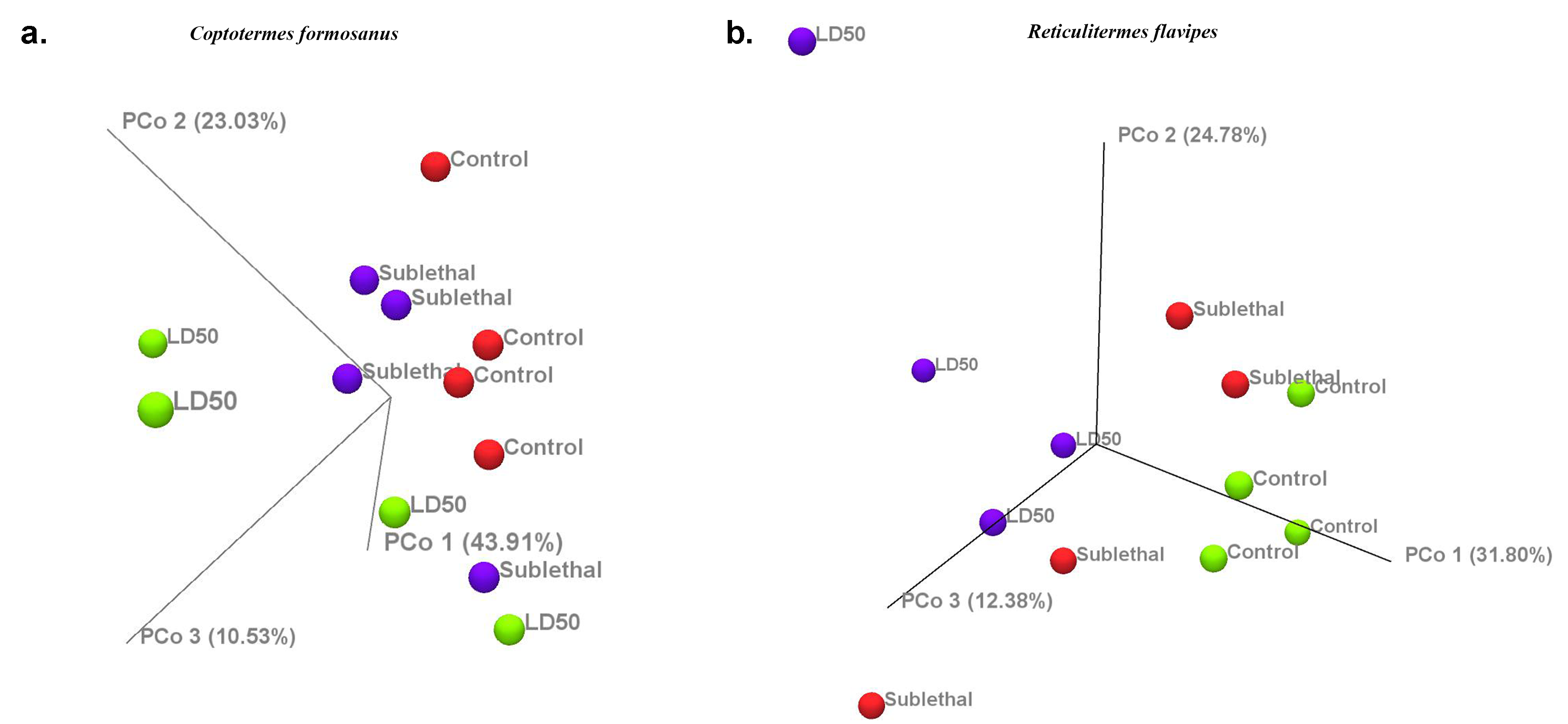

Supplement: Supplementary Figure 4 — PCoA analysis. (a) PCoA plot representing the beta diversity based on Bray-Curtis distance in eugenol-treated and untreated control C. formosanus samples. (b) PCoA plot representing the beta diversity based on Bray-Curtis distance in eugenol-treated and untreated control R. flavipes samples. LD50, lethal dose of eugenol. [file Image_4.tif]

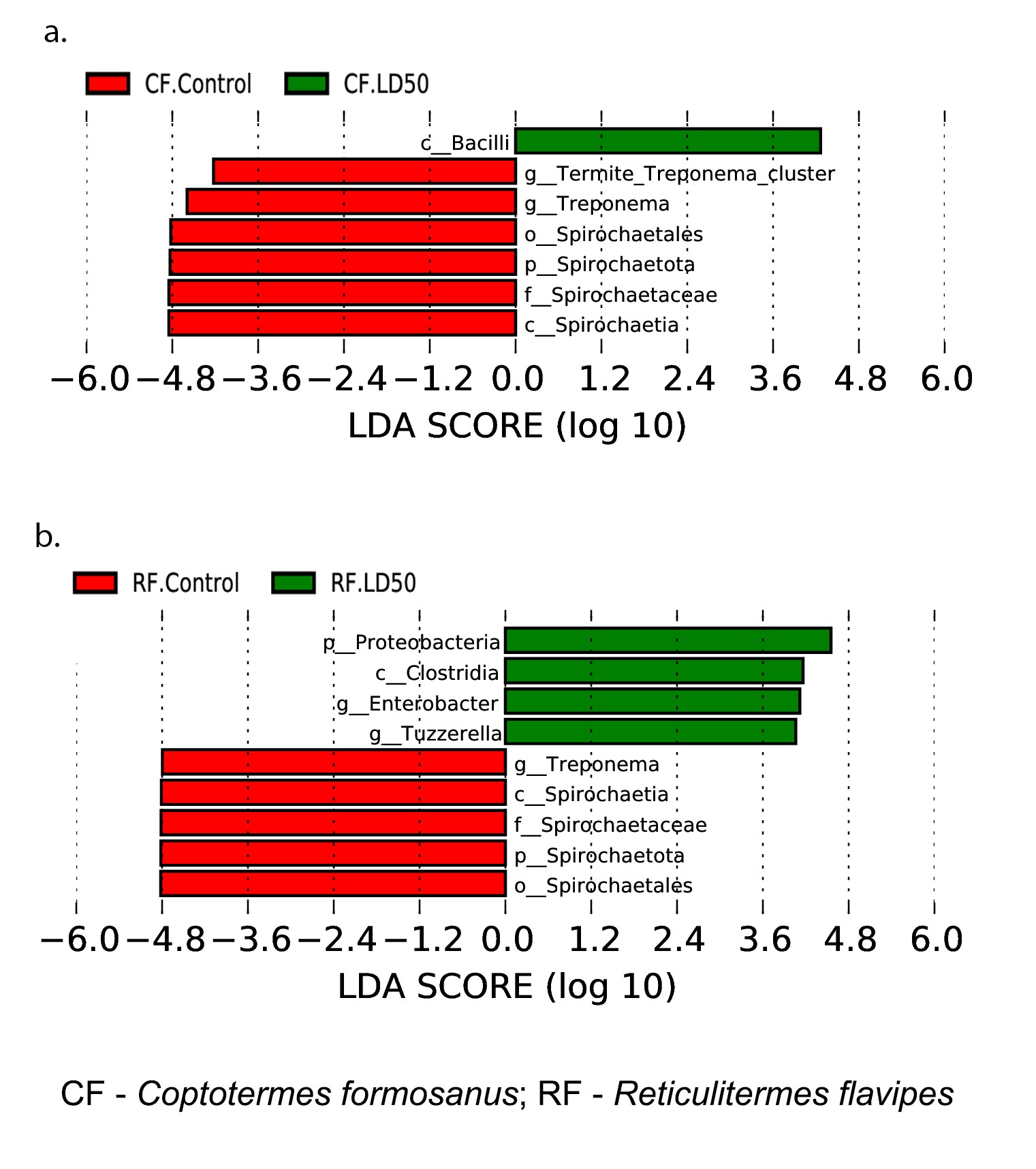

Supplement: Supplementary Figure 5 — LEfSe analysis. Histogram representing the LDA scores depicting the significantly abundant bacterial communities. (a) Eugenol-treated and untreated C. formosanus samples, (b) eugenol-treated R. flavipes, and the untreated termite control. CF- C. formosanus; RF- R. flavipes; LD50- lethal dose of eugenol. [file Image_5.jpeg]

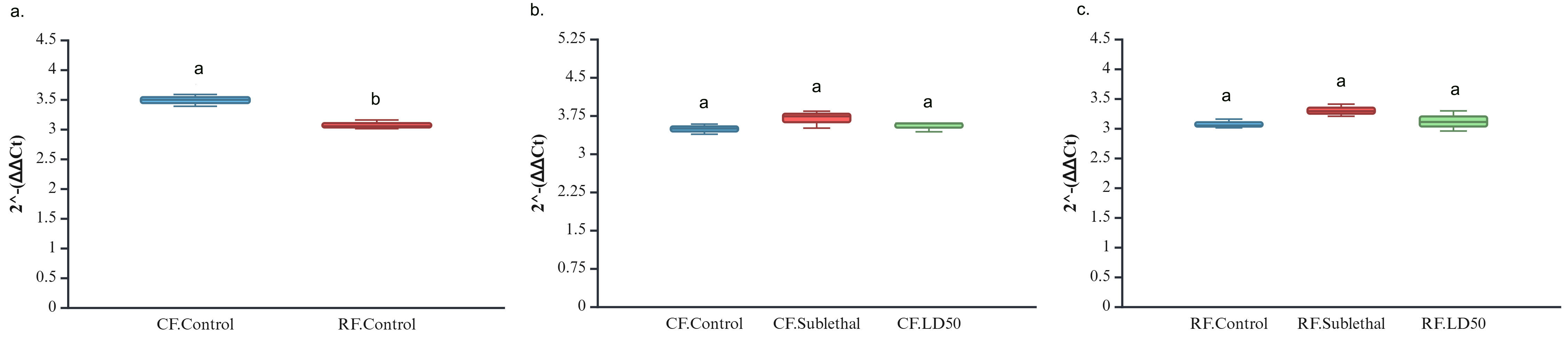

Supplement: Supplementary Figure 6 — qPCR assay. Bacterial 16S rRNA gene copy numbers in C. formosanus and R. flavipes in different conditions, such as (i) between two subterranean termite species, (ii) eugenol-treated and untreated C. formosanus samples, and (iii) eugenol-treated and untreated R. flavipes samples. Data was log-transformed for better visualization. The statistical comparison was performed using the one-way ANOVA with Tukey multiple comparisons test. [file Image_6.jpeg]

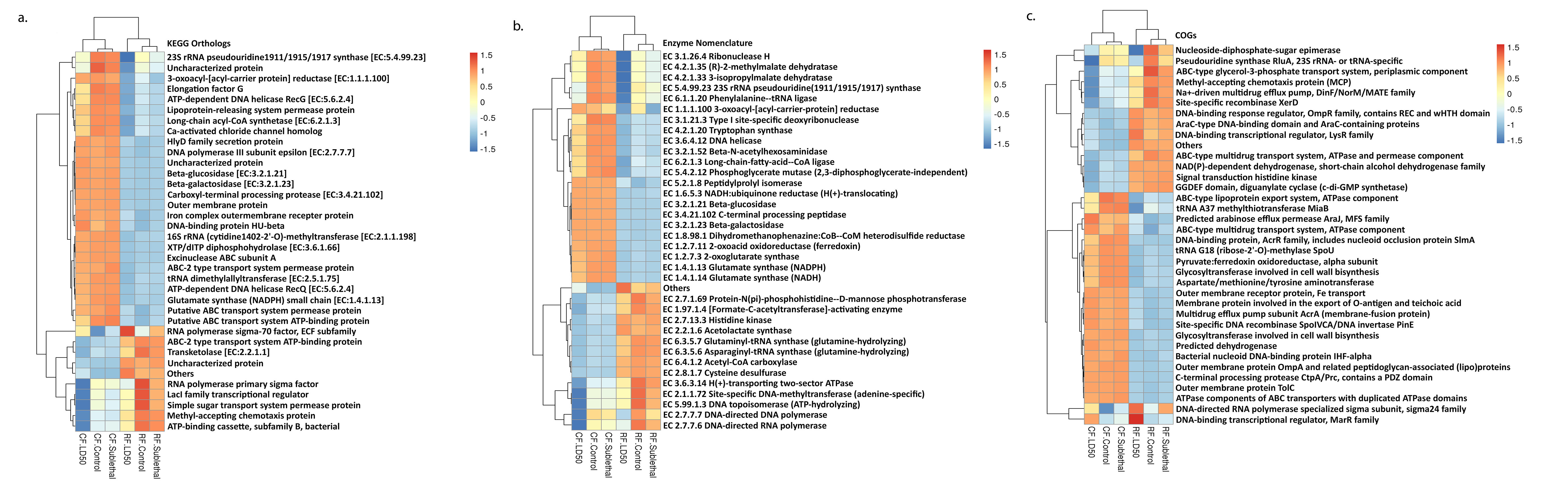

Supplement: Supplementary Figure 7 — Functional prediction using PICRUSt2. Heatmap illustrates the putative bacterial functional contribution based on (a) KEGG, (b) EC, and (c) COG databases. [file Image_7.jpeg]

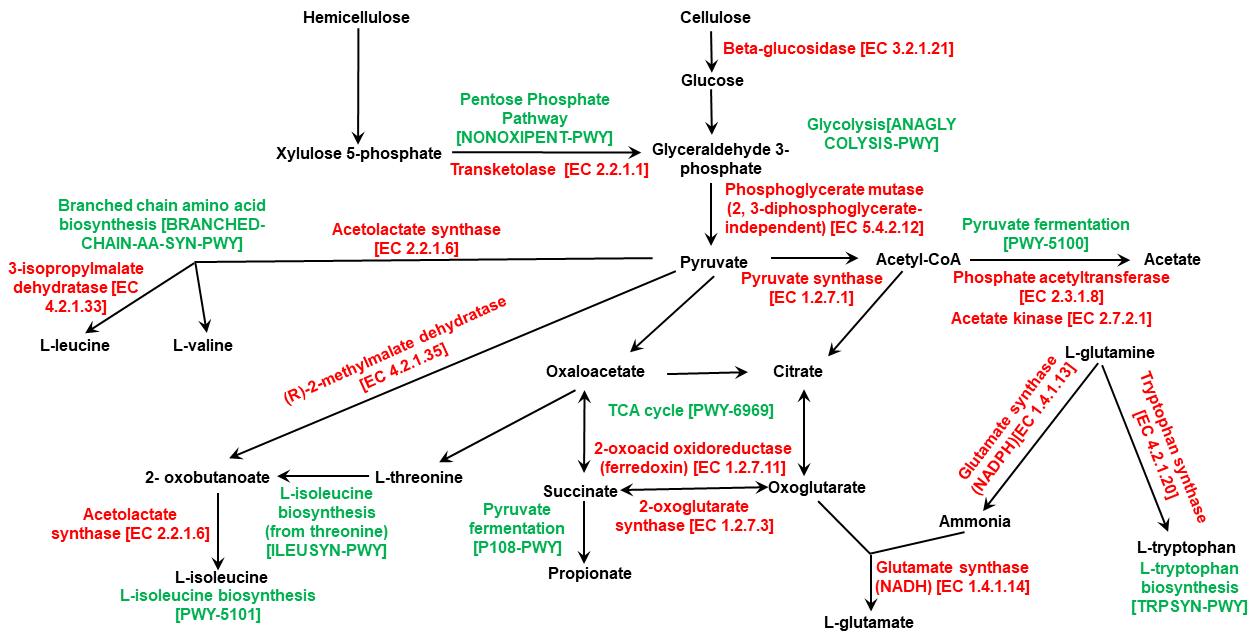

Supplement: Supplementary Figure 8 — Schematic diagram of the enzymes predicted using PICRUSt 2 that are involved in the wood degradation pathway. [file Image_8.tif]
